# Exponentially increasing microplastic accumulation in an urban estuary: insights from the Narragansett Bay, Rhode Island seafloor

**DOI:** 10.1007/s11356-025-37295-2

**Published:** 2025-12-19

**Authors:** Victoria M. Fulfer, John P. Walsh, David Reide Corbett

**Affiliations:** 1 Graduate School of Oceanography, University of Rhode Island, Narragansett, RI USA; 2 The 5 Gyres Institute, Santa Monica, CA USA; 3 Coastal Studies Institute, Eastern Carolina University, Wanchese, NC USA

**Keywords:** Microplastics, Marine pollution, Sediment, Coastal sediment, Plastic deposition, Estuarine pollution

## Abstract

Narragansett Bay, RI, has experienced decades of anthropogenic impacts and as a result has been a hotspot for pollution research. Most recently, microplastic pollution is of increasing concern, with data showing high levels of microplastic contamination on shorelines and in surface sediments. Plastic production worldwide has been increasing exponentially since the 1950s, and this system-wide chronologic study uses seabed and marsh sediment cores to show that plastic pollution in our coastal systems reflects the same exponential trend. Using ten sediment cores, 20–42 cm in length, ranging from outside the city of Providence in the northern estuary to 30 km offshore in Rhode Island Sound, we find exponentially increasing microplastic (63 µm–5 mm) concentrations from the 1940s to the present day. All sites show increasing diversity in plastic polymer types, colors, and morphologies over time. Marsh sediments contained microplastic concentrations ten to fifty times higher than the nearby seabed and have been accumulating microplastics at four times the rate over the past century, raising concern for the benthic and aquatic biota that rely on these important habitats. Today, nearly all sediment sites (9 out of 10) have exceeded thresholds of harm for benthic organisms. Model results suggest anywhere between approximately 2300 and 3000 tonnes of microplastics have accumulated in the Narragansett Bay seabed sediments over the past century. With few wide-scale mitigation measures in sight, these results should serve as a warning that without action, the pollution of our coastal systems will continue to increase exponentially.

**Supplementary Information:**

The online version contains supplementary material available at 10.1007/s11356-025-37295-2.

## Introduction

Plastic production has been increasing exponentially worldwide since the 1950s, reaching 413.8 million tonnes in 2023 (Geyer et al. 2017; Plastics Europe 2024). Beginning with Parkesine in the mid-nineteenth century, the variety of plastics, molding methods, and uses increased dramatically in the 1920s and 1930s, and this diversity was showcased at the “World of Tomorrow” in the 1939 World’s Fair (Clark 2015; Geyer 2020). Today, rivers deliver an estimated 5.1 to 24.9 million tonnes of mismanaged plastic waste composed of dozens of different polymer types to coastal regions (Borrelle et al. 2020; Jambeck et al. 2015) and the ocean each year (Meijer et al. 2021). Once in the environment, plastic materials become more brittle and break down into particles called microplastics (MPs), which range from 1 µm to 5 mm in diameter (Cózar et al. 2014; Song et al. 2017). MPs are an array of synthetic polymers found in the environment in the form of films, fibers, fragments, pellets, or foams (Helm 2017; Lusher et al. 2020). Due to their small size and transport through environmental systems, MPs have been found in every environment on Earth, including the deep sea, Arctic sea ice, coastal sediments, and throughout the global ocean (Andrady 2011; Barnes et al. 2010; Cole et al. 2011; Eriksen et al. 2014; Harris et al. 2023; Woodall et al. 2014). Once in the environment, microplastics have been shown to be ingested by hundreds of marine species with a range of negative impacts (Cole et al. 2011; Mazurais et al. 2015; Rochman et al. 2014; Rochman et al. 2013a, b; Sussarellu et al. 2016). Benthic invertebrates (e.g., bivalves, sea urchins, polychaetes, crabs) are of particular concern for MP ingestion due to their potential habitation in polluted sediments and their filter, suspension, or deposit feeding modes (Van Cauwenberghe et al. 2015).

MPs pose a pronounced global threat to coastal and marine ecosystems, with the impact anticipated to be amplified in the coastal zone, where terrestrial inputs are high and where hydrodynamic, biochemical, and sedimentary processes allow for increased plastic accumulation (Egea et al. 2023; Gray et al. 2018; Harris 2020). While many plastic particles are buoyant upon entry into the marine environment, biofouling and other processes increase their sinking rates over weeks to months, causing even initially buoyant particles to sink to the seafloor (Kaandorp et al. 2020; Kvale et al. 2020; Lobelle et al. 2021; Martin et al. 2022; Ye and Andrady 1991). Recent studies suggest that 70–99% of plastics entering the marine environment are deposited on shorelines and in marine sediments (Frias et al. 2016; Law 2017; Onink et al. 2021).

MPs have been observed in the benthic environment as early as the 1970 s (Gregory 1977; Shiber 1979), and sedimentary strata can serve as long-term sinks for microplastics (Cózar et al. 2014; Morét-Ferguson et al. 2010). Recent studies have focused on determining a temporal record of MPs in various environments using sediment cores from lakes, rivers, estuaries, and the open ocean (Brandon et al. 2019; Dahl et al. 2021; Li et al. 2020; Martin et al. 2022). The focus of this study was to analyze sediment cores throughout Narragansett Bay, RI, and reconstruct the microplastic pollution history of this important urban estuary.

Narragansett Bay, RI, USA, is the largest estuary in New England, measuring about 40 km from the mouth to the head of the Providence River, with a mean water depth of 8.7 m. This bay was once considered the most industrialized estuary in the world (Kutcher 2009) after becoming the birthplace of the Industrial Revolution in America in 1790 (Conrad 1995). Due to high industrial inputs and dense urban populations, the water and sediments of Narragansett Bay have been subject to anthropogenic contamination including nitrogen and phosphorus inputs, heavy metals, petroleum hydrocarbons, and now, microplastics (Fulfer and Walsh 2023; Hartmann et al. 2005; Latimer and Quinn 1996; Nixon 1995; Santschi et al. 1984). Nearly all freshwater and urban pollutants enter the estuary from rivers, runoff, and sewage treatment plants located on the northern shorelines, with very little freshwater and sediment input in the southern estuary (Nixon 1995; Pilson 1985).

Many studies have shown that organic pollutants, heavy metals, and other contaminants in Narragansett Bay display a decreasing spatial gradient from the head of the Bay to the mouth, and an increasing trend through time until mitigation measures were implemented (Bricker 1993; Goldberg et al. 1977; Nixon and Fulweiler 2012). Two previous studies have shown high modern-day levels of microplastic concentrations in shoreline and seafloor sediments of Narragansett Bay (Cashman et al. 2022; Fulfer and Walsh 2023). No state-wide mitigation measures for plastic pollution have been implemented in Rhode Island to date, but local measures, such as bag bans, are being implemented. It is important now to establish a historical context for the levels of MP pollution around the Bay and provide a modern-day baseline from which future conditions can be compared. Such information may be used to motivate action and assess the effectiveness of mitigation and clean-up efforts. Additionally, Narragansett Bay has been used as a burial sink for hard-to-remove pollutants such as heavy metals, with sediments being deliberately left undisturbed to allow contaminants to be sequestered over time (Nixon et al. 1986). Similarly, MPs that accumulate in seafloor sediments could be resuspended into the water column, and thus the marine food web, if disturbed during dredging and other activities. An understanding of the long-term burial rates of MP is necessary to assess management strategies for plastics and related chemical contaminants.

This study represents the first system-wide chronologic study of microplastic accumulation on the U.S. East Coast. The goals of this study were to (1) measure the accumulation of microplastic pollution across Narragansett Bay through time using sediment cores, (2) determine if microplastic accumulation rates differ between seafloor and marsh sediments, and (3) assess geographical distributions of MP load in estuarine sediment. Our findings contribute to the overall understanding of historical trends and depositional patterns of MP pollution, shed light on potential sources, and indicate which environments may best serve as sinks for these emerging pollutants.

## Methods

### Sampling

**Fig. 1:**
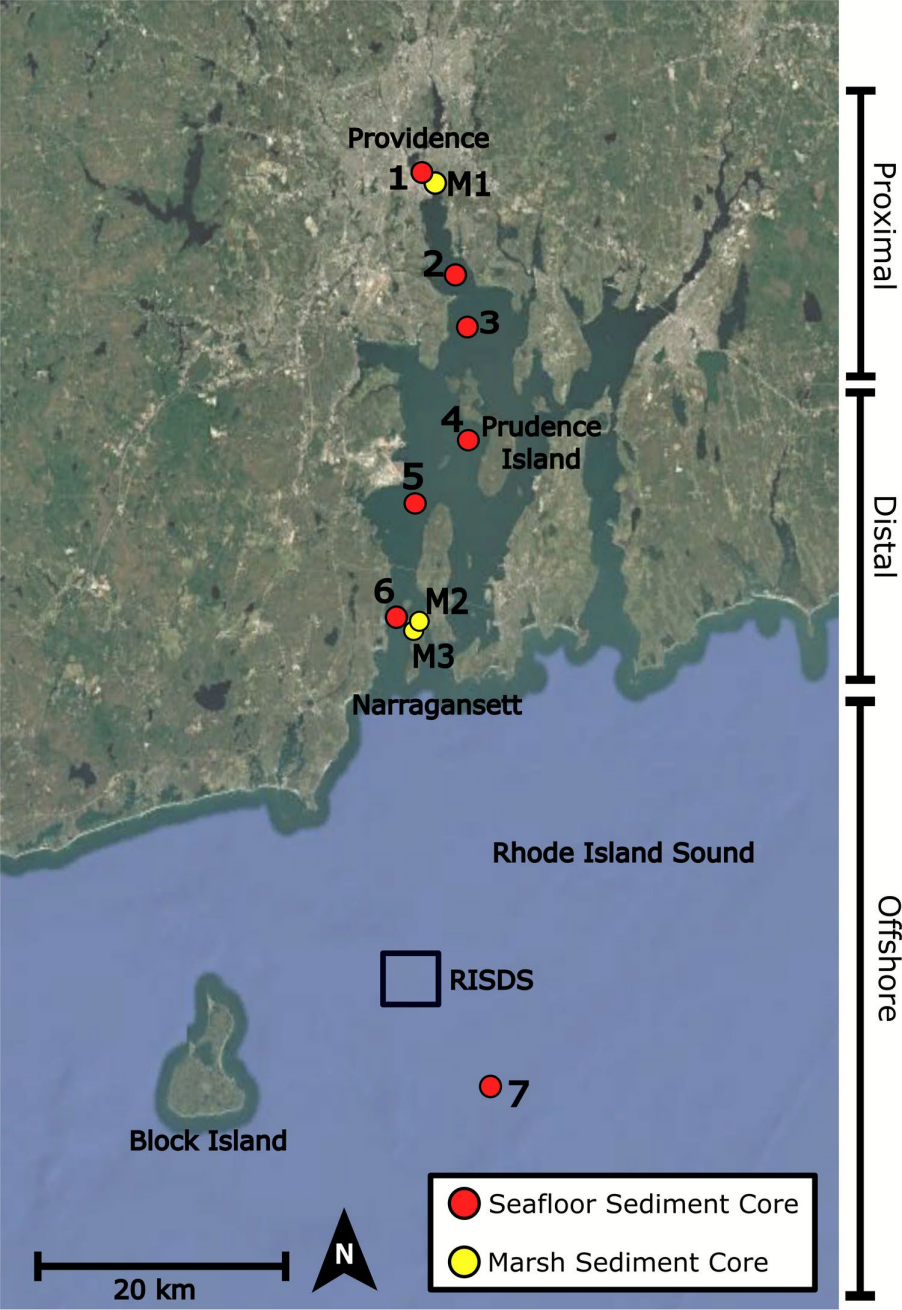
Locations of seafloor (red circles) and marsh (yellow circles) sediment cores in Narragansett Bay, RI, and Block Island Sound. Seafloor core sites are numbered from north to south (1–7) and marsh sites are denoted by an M (M1–M3). Sites are located with increasing distance from the city and, to add distinction, are situated in either the Proximal, Distal, or Offshore Zones. Rhode Island Sound Disposal Site (RISDS) has been used to dump dredge material since the 1960s. Basemap derived from Landsat 8 image courtesy of the U.S. Geological Survey, Google Earth Pro, V 7.3.6.10201, Image Landsat/Copernicus, December 31, 2020

For this study, Narragansett Bay has been split into two zones: the Proximal Zone is within 17 km of the city of Providence, or any area north of the northernmost tip of Prudence Island, and the Distal Zone extends south of this point to Narragansett, at the mouth of the Bay (Fig. 1). Gravity cores with a relatively undisturbed sediment-water interface were collected throughout Narragansett Bay from the *R/V Cap’n Bert* using a check-valve Universal Corer from Aquatic Research. Seven sites were sampled ranging from the Lower Providence River to offshore Narragansett, RI (Fig. 1). Core lengths varied from 20 to 42 cm. Core diameters were 7 cm for Sites 1–6 and 10 cm for Site 7. Seafloor Sites 1–3 (May 2021) are located in the Proximal Zone relative to Providence (Fig. 1), and seafloor Sites 4–6 (May 2022) are located in the Distal Zone. One multi-core, Site 7, was collected offshore Block Island, RI from the *R/V* Endeavor in September 2019. All cores were encased in PVC and kept upright and refrigerated until processing. The PVC core casings are a potential but, in this case, unavoidable source of microplastic contamination in this study. Sediment was removed from the PVC casing as soon as possible after sampling.

Site 1 is located in the channel of the Lower Providence River, just over 2 km from the city center of Providence, RI (Fig. 1; Supplemental Fig. 1). This area of Narragansett Bay is lined with industrial activities today and was so historically. Through inadvertent losses (during production or transportation of materials or waste) or deliberate actions (e.g., littering) as well as wastewater treatment facilities, there is a wide array of potential sources of microplastic pollution. This region of Narragansett Bay has an almost completely hardened shoreline with riprap on the western shore, and some small areas of fringe marsh on the eastern shore. Site 2 is located offshore Conimicut Point (Supplemental Fig. 2), while Site 3 is located 5 km to the south, offshore Rocky Point State Park (Supplemental Fig. 3), both adjacent to extensively developed land.

Site 4 is located off the western shore of Prudence Island, just south of the Jenny’s Creek Shellfish Management Area (Fig. 1; Supplemental Fig. 4). Site 5 is seaward of the Port of Davisville, another area with active cargo shipping and widely hardened shorelines (Supplemental Fig. 5). Located between the University of Rhode Island’s Bay Campus and Dutch Island, Site 6 is closest to the mouth of Narragansett Bay (Supplemental Fig. 6). Finally, Site 7 was cored in Rhode Island Sound, 25 km southeast of Point Judith (Supplemental Fig. 7).

Three marsh cores were collected using a Russian Corer in May 2022. Approximately 50 cm half cores, 6.5 cm diameter, were collected at Watchemocket Cove (M1), the outer zone of Fox Hill Marsh (M2), and the inner zone of Fox Hill Marsh (M3) (Fig. 1; Supplemental Figs. 8 and 9). The inner zone receives less tidal input and less frequent flushing from the nearby inlet than the outer zone. Watchemocket Cove is located southeast of Site 1, in a small embayment attached to the Lower Providence River, while Fox Hill Marsh is located in the Distal Zone of the Bay, in a small embayment of Conanicut Island.

Seafloor (Sites 1–6) and marsh (Sites M1–M3) cores were sectioned into 2 cm intervals and the offshore core (Site 7) was sectioned into 1 cm intervals. Half of each section was retained for microplastics analysis in pre-rinsed glass jars, while the other half was retained in Whirlpak bags for grain size and ^210^Pb analysis.

### Sediment properties

A subsample of each section was homogenized and dried at 65° C for 24 h, with mass recorded before and after drying. The calculated water content was used to determine the dry weight of each sampled section. Grain size distributions of homogenized samples were measured using a combination of sieving (> 2 mm, gravel component) and a Mastersizer 3000 particle laser scanner (< 2 mm). Each sample was measured in triplicate to obtain a robust average distribution per sample. For each sample, 100 particle size bins were reported by the Mastersizer 3000, which were then condensed into four size bins corresponding to clay (< 2 µm), silt (2–63 µm), sand (63 µm–2 mm), and gravel (> 2 mm) to visualize grain size distributions. Sediment particle sizes ranged from 0.3 µm to 3.5 mm.

Radioisotope profiles of ^210^Pb, with a half-life of 22.3 years, can be an effective tool for determining the accumulation rate of muddy sediment over the past 100 years (Corbett and Walsh 2015). Down-core sediment sections were analyzed by alpha spectroscopy following the methodology of Corbett and Walsh (2015). In short, 1.0 g of dried sediment was spiked with 1.0 mL ^209^Po of a known activity level and digested with a strong acid (8 N HNO_3_). The solution was then electrodeposited onto nickel discs in an acid solution, and the activity levels of ^209^Po and ^210^Po were measured on an EG&G Octet alpha spectrometer.

Sediment accumulation rates were determined for each core using a constant flux-constant sedimentation (CFCS) model (Appleby and Oldfield 1978) below the mixed layer (5 cm). The CFCS model is often referred to as the “Simple” model because it assumes constant flux and sedimentation over the period examined. Given the relatively modest amount of activity data, and consistent dry bulk density observed in 7 out of 9 cores, we believe the CFCS model provides a conservative and reasonable geochronology. The CRS and CIC models require confidence in the inventory and the initial concentration of ^210^Pb, respectively, to allow determination of variable sedimentation rates (Corbett and Walsh 2015). Excess ^210^Pb activity (total ^210^Pb − supported ^210^Pb) was plotted as a function of sediment depth on a semi-logarithmic scale and fit with a linear regression model. The linear regression represents the relationship between the natural log of excess ^210^Pb activity and depth, where the slope (*m*) describes the rate of ^210^Pb decay with depth. Using the CFCS model, sediment accumulation rates (*S*) were derived using the equation:

$$S= -m\lambda$$

where λ is the ^210^Pb decay constant (0.03114 year^−1^).

### Microplastic extraction and analysis

Microplastic particles larger than 63 µm were extracted from sediment samples using methods described in full in Fulfer and Walsh (2023), which have an average extraction efficiency of 92%. In short, 10–50 g of wet sediment was sieved at 62.5 µm to remove the fine-grained fraction. Remaining sediment was mixed in a dense solution of sodium iodide (NaI; 1.8 g cm^−3^) to extract plastic particles from sediment particles. This density extraction was completed in triplicate for maximum extraction efficiency (Fulfer and Walsh 2023). The supernatant was filtered, rinsed with deionized water, and any particulate organic matter removed using 10% H_2_O_2_ + 10% HCl at 60 °C for 24 h. The final digested solution was filtered onto 47-mm, 5-µm pore size PTFE filters and stored in glass petri dishes for analysis.

Filters were examined for microplastic particles using light microscopy under an AmScope dissecting microscope at ×40 zoom. NIGHTSEA royal blue (440–460 nm excitation) fluorescence viewed through a 500-nm longpass emission filter (NIGHTSEA SFA Stereo Microscope Fluorescence Adapter) was used to help locate plastic particles on the filter. All suspected plastic particles were counted and their morphology (e.g., fragment, fiber, film) and color recorded.

### Microplastic polymer identification and quantification

Approximately 50% of counted particles underwent FT-IR analysis to verify as plastic or non-plastic. Following visual counting, particles were washed from filters onto 25-mm, 0.45-µm pore size silver filters (Sterlitech) and allowed to dry for 24 h. Verification of suspected microplastic particles and determination of chemical composition were completed using a linear-array µFT-IR spectroscopic imaging system (Spectrum™ 3 and Spotlight™ 400, PerkinElmer). The entire filter area (17 mm × 17 mm) of 40 samples containing mixtures of suspected MP particles, organic matter, and fine sediment grains was scanned in reflectance mode over a spectral range of 750–4000 cm^−1^ at 8 cm^−1^ spectral resolution and 25 µm pixel resolution applying 4 co-added scans (Pabortsava and Lampitt 2020). Following µFT-IR spectroscopy scanning of the filter area, polymer identification was performed based on the methods of Pabortsava and Lampitt (2020) and using the PerkinElmer Spectrum™ IMAGE and Spectrum™ 10 softwares. In short, a chemometric principal component analysis (PCA) was used to analyze the chemical composition of the entire spectral image map and collect spectra from each PCA variation (score). All collected individual spectra were compared against spectra in the reference polymer libraries (e.g., the spectra database from S.T. Japan-Europe GmbH, Germany/Japan), and spectra with a hit quality > 0.75 were accepted as verified microplastic particles (Supplemental Fig. 10). The best quality spectra were selected and used as reference spectra to map against every pixel in the infrared image, generating maps of all particle types. All mapped particles were then exported as image files. Using FIJI ImageJ image software, particle counts were conducted (Erni-Cassola et al. 2017; Schindelin et al. 2012).

Shannon diversity (H′) was calculated for microplastic particle color, morphology, and polymer type for each site-year interval. Color categories were assigned based on visually identified hues, particle types were grouped into standard morphological classes (e.g., fibers, fragments, films, foams), and polymers were identified by µFT-IR. For each category set, counts or amounts were summed within each site and depth layer and reshaped into site-by-year matrices. Shannon diversity was calculated as H′ = − Σ *pᵢ* ln(*pᵢ*), where *pᵢ* is the proportional abundance of each category within a sample. Richness was defined as the number of categories present (*S*), and evenness was calculated as *J* = H′/ln(*S*). Diversity metrics were computed separately for color, particle type, and polymer composition and were also evaluated as combined polymer–type–color classes by concatenating category labels. Temporal trends were assessed using linear regression of H′ against year for each site, and significance was evaluated using α = 0.05.

Once quantification of verified microplastics was complete, microplastic concentrations per sample were calculated using the mass of sediment extracted and microplastic count (MP particles per kg dry weight (DW) sediment). The salt-corrected dry bulk density of each section was calculated from the dry mass, core section volume, porewater density, and assumed particle density of 2.65 g cm^−3^. Seabed sediments in Narragansett Bay are a mixture of sand, silt, and clay (McMaster 1960) and most of the sand is low-density quartz (2.65 g cm^−3^) (McMaster 1962). This density is the middle range of common minerals and rocks; therefore, it is used to calculate the bulk density of sediment. Down-core sediment mass accumulation rates (MAR; g cm^−2^ year^−1^) were calculated as the product of the dry bulk density (g cm^−3^) of each interval and the modeled sediment accumulation rate (cm year^−1^) (Ryan-Mishkin et al. 2009). Using the MAR and the microplastic concentration at each depth, microplastic accumulation rates (MPAR) through time were determined.

A basic, back-of-the-envelope, linear model of microplastics storage in Narragansett Bay sediment was calculated. Sediment cores were collected in mud-dominated areas where fine sediment is accumulating (McMaster 1960). The model was applied only to seafloor areas of Narragansett Bay where fine-grained sediment is accumulating, based on samples collected and maps created by McMaster (1960). Using linear relationships between distance from the urban center of Providence (latitude) and MPARs in Proximal and Distal Zones, estimates of MP accumulation for each decade and over the past century were calculated (see Supplemental Methods; Supplemental Fig. 16). MPARs for each decade were converted from particles m^−2^ year^−1^ to g m^−2^ year^−1^ using a conservative average mass per microplastic particle > 63 µm (58.6 µg) reported in Fulfer and Walsh (2023).

### Contamination control

Contamination control, blank corrections, and quantification of uncertainties are all particularly important during MP analysis. All steps involving sample processing and analysis were performed in a laminar flow cabinet. All laboratory ware used was made of glass or stainless steel and cleaned thoroughly prior to use with pre-filtered water at 0.45 µm. During sample processing, containers were kept closed or covered whenever possible. All chemicals used for sample processing were also filtered through a 0.45-µm silver filter (Sterlitech) prior to use.

Three types of blank samples were collected and analyzed during the processing of each sample batch. Air blanks were collected using a filter exposed in an open glass petri dish in the laminar flow hood during sample processing. Blanks of 1 L of MilliQ water were also collected on silver filters during each sample batch. Procedural blanks were collected by running through the entire microplastic extraction procedure without the presence of sediment, and then analyzing the collected particles.

Mean blank values for the number of particles found in all three blank types were used to calculate the limit of detection (LOD) and limit of quantification (LOQ), which are currently the most reliable for microplastics data (Dawson et al. 2023). The LOD was defined as ×3.3 the standard deviation and the LOQ was defined as ×10 the standard deviation (AOAC INTERNATIONAL 2016; Johnson et al. 2020). The LOD value was calculated for each particle morphology (e.g., fragment, fiber, film, foam, bead) and as a total count LOD for all morphologies combined. The LOD value was subtracted from all samples based on the particle morphology as a blank correction (Dawson et al. 2023; Tsering et al. 2022). The LOD values ranged from 1.9 to 3.8 particles and LOQ ranged from 5.7 to 11.5 particles across analytical batches. Any samples with particle counts less than the LOD value for that analytical batch were not considered for analysis and were assumed to have an MP concentration of 0 particles kg^−1^. Samples with counts greater than the LOD but below the LOQ are included in analyses and indicated in figures with an asterisk (*). Samples with microplastic counts greater than the LOD and greater than the LOQ were considered detected and quantifiable.

## Results

### Sedimentary environment

**Fig. 2:**
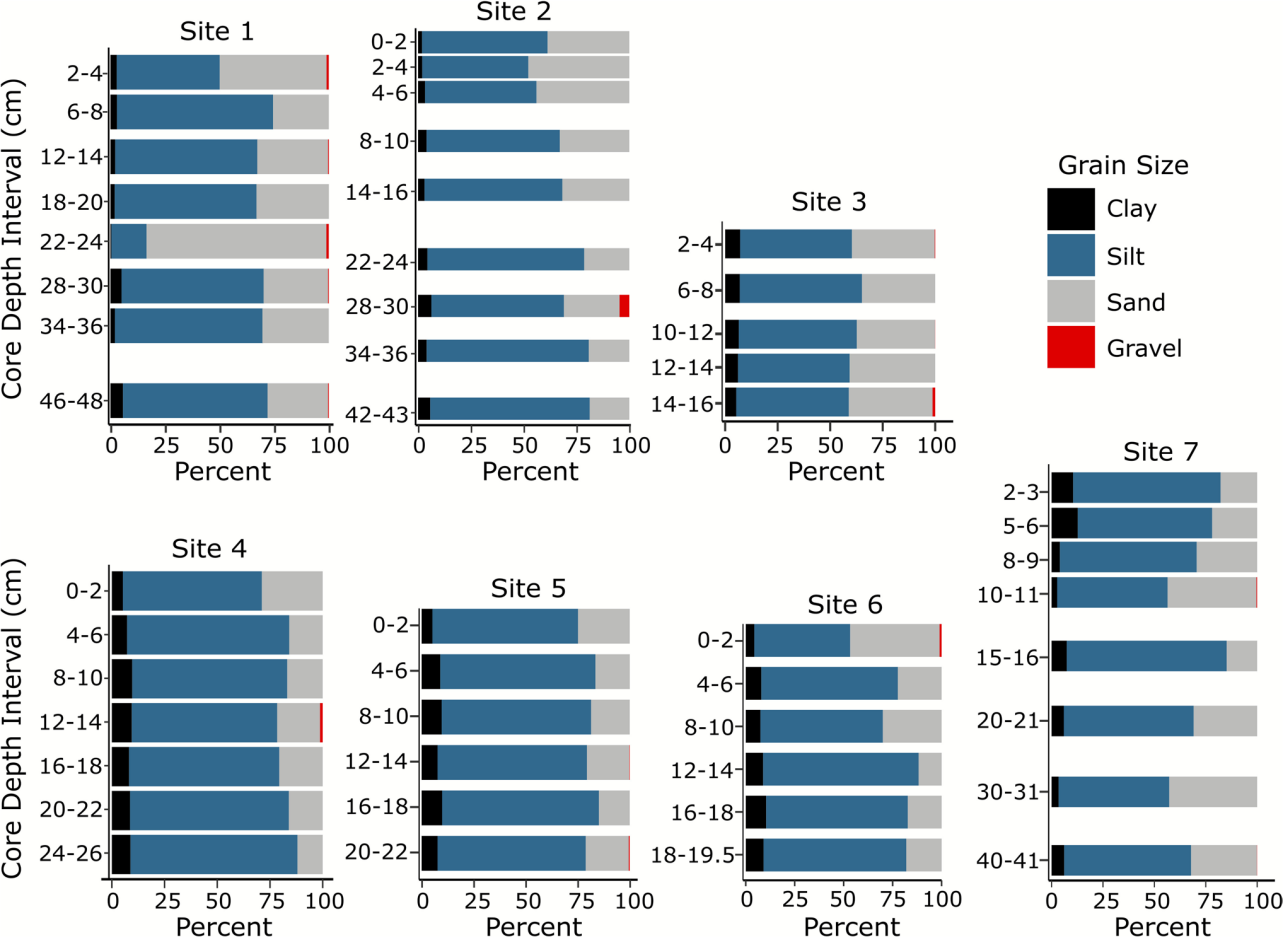
Grain size compositions for seafloor cores. Silt (blue) dominated at all sites and through time, with sand (gray) varying through time and space

The cores collected were dominantly composed of muddy sediment (e.g., silt and clay) and show relatively modest vertical changes except for a coarsening-upward trend in Core 2 and a sand layer at 22 cm in Core 1 (Fig. 2). Sediments of Narragansett Bay are primarily derived from glacial and post-glacial deposits with accumulating modern fine-grained sediments generally found in deeper areas (Santschi et al. 1984). The cores for this study were purposefully collected in mud-rich zones mapped by McMaster (1960). More specifically, all sediment layers across all cores in this study were silt-dominated, ranging from 54.8 ± 2.2 to 73.6 ± 3.8% (*n* = 49). Gravel fractions never exceeded 5% and averaged 0.2 ± 0.7% across all samples analyzed. Average mud composition across all depths in cores increased down system, with clay fractions ranging from 2.9 ± 1.7% in the Proximal Zone to 8.2 ± 1.5% in the Distal Zone, and 6.6 ± 3.5% at the offshore core in Rhode Island Sound (Site 7). In concert with the mud fraction changes, the measured sand fraction decreased down system, being higher in the Proximal Zone (average 39 ± 19%) than in the Distal Zone (19 ± 5.4%). However, it is important to emphasize that strong lateral gradients were mapped by McMaster (1960) reflecting the estuarine channel morphology controlling post-glacial mud accumulation.

Sediment grain size distributions varied subtly down-core in many of the sediment cores. Site 1 exhibited the most variable down-core grain size compositions, likely due to the highly developed and disturbed nature of this portion of the estuary, including dredging in this area (USACE & INSPIRE Environmental 2020). Sites 2, 4, and 6 all exhibited a nearly 20% (Site 2) to 30% (Sites 4 and 6) increase in sand in the top ~20 cm. Site 7, located offshore, saw the opposite trend, with increasing mud at the top of the core. Sites 3 and 5 were relatively constant vertically, with less than 10% change in any grain size fraction throughout the entire core.

**Fig. 3:**
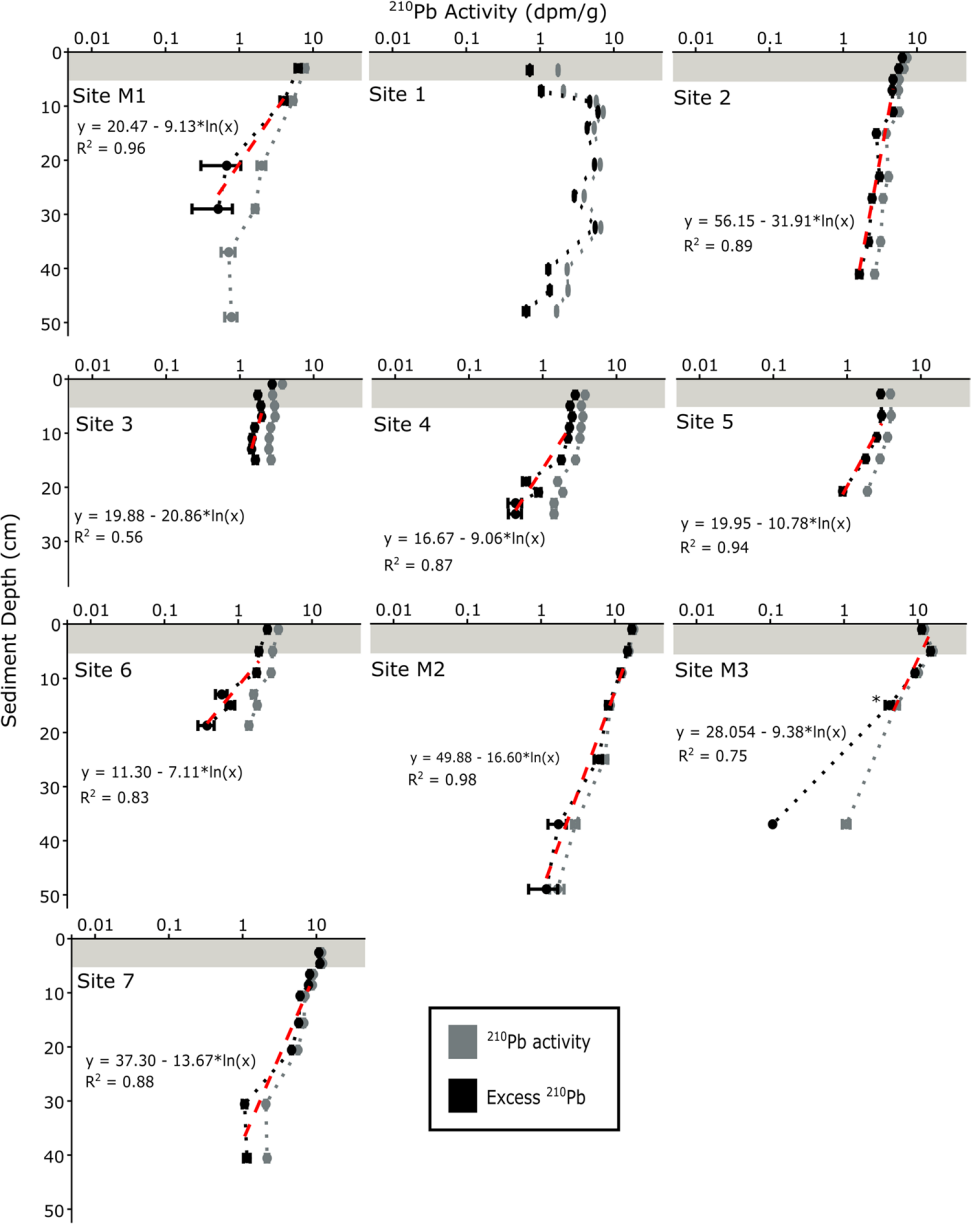
Sediment accumulation rates were calculated from down-core ^210^Pb radioisotope activity profiles (dmp/g). Based on the ^210^Pb activities, a bioturbated surface mixed layer of sediment was determined to be 5 cm deep (gray shaded zone). Red dashed lines denote the logarithmic regression of excess ^210^Pb activities with sediment depth. For Site M3, an asterisk (*) denotes the deepest point used for sediment accumulation rate calculations. A non-steady-state profile in Site 1 precluded an accumulation rate determination

Linear sediment accumulation rates were calculated for nine of the ten cores collected in this study (Fig. 3); a non-steady-state profile in Site 1 precluded an accumulation rate determination. Sediment accumulation rates in seafloor cores ranged from 0.99 ± 0.13 cm year^−1^ at Site 2 to 0.22 ± 0.05 cm year^−1^ at Site 6 near the mouth of the Bay, with accumulation rates generally decreasing from north to south. Site 7, located offshore, had a sediment accumulation rate of 0.43 ± 0.06 cm year^−1^. Marsh sediment accumulation rates ranged from 0.20 ± 0.01 cm year^−1^ (Site M3) to 0.5 ± 0.04 cm year^−1^ (Site M2). Several variables can introduce error in radioisotope sediment dating. In addition to measurement-related errors in activities, environmental factors can affect sediment data and derived sediment accumulation rates, such as variable radioisotope flux and sediment supply due to changing input or scavenging as well as biological or physical mixing (Barsanti et al. 2020; Palinkas and Nittrouer 2007). Potential errors and challenges are discussed further by Corbett and Walsh (2015).

Based on the sediment accumulation rates and a mixed layer depth of 5 cm, the age of each sediment layer was calculated. Calculated ages for the deepest sediments in the cores ranged from 2003 in the shortest core (Site 3, 17 cm) to 1935 in the longest core (Site 7, 41 cm). Marsh core sediments spanned a larger age range, reaching as old as nearly 1900 (Site M3).

### Microplastic depositional patterns

**Fig. 4:**
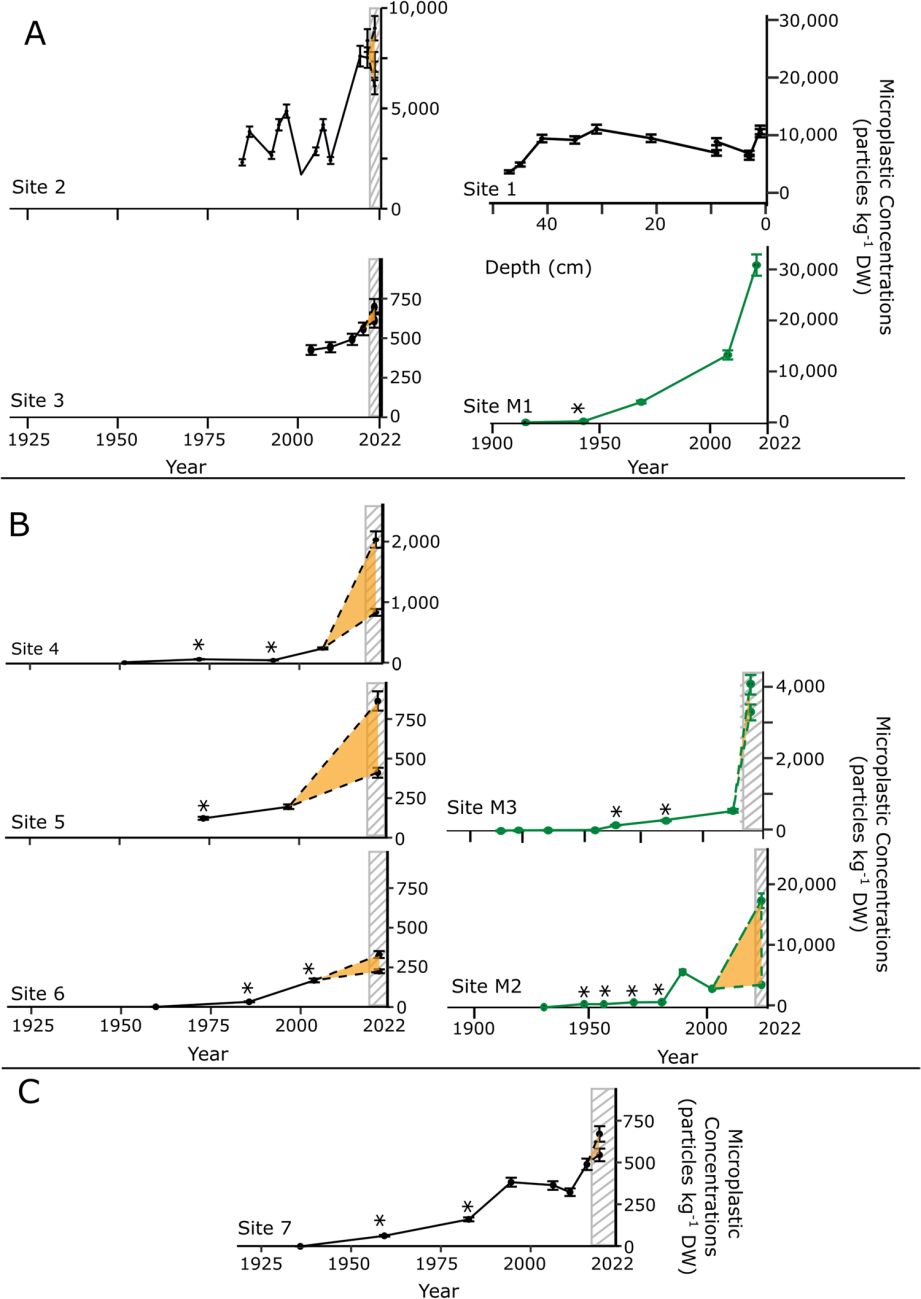
Microplastic concentrations through time for all seafloor (black dots and lines) and marsh (green dots and lines) sites in the Proximal (**A**), Distal (**B**), and Offshore (**C**) Zones of Narragansett Bay. Error bars represent analytical error. At some core depths, replicate extractions were completed and all data points are shown, with dotted lines and shaded orange zones representing the range between the next core section and the lowest and highest replicate concentrations. The hatched gray zone represents the sediment mixed layer depth, which was estimated to be 5 cm at all sites. Samples with counts greater than the LOD (detectable) but less than the LOQ (not quantifiable) are included in analyses and indicated in figures with an asterisk (*). Data falling earlier in time than the data denoted with an asterisk (*) have a microplastic concentration of 0 particles kg^−1^ DW

Microplastic concentrations were calculated for all collected sediment cores at regular depth intervals after particle counts were blank corrected using the LOD. One sample fell below the LOD and was adjusted to a count of 0. Out of the 70 counted samples, 16 fell above the LOD but below the LOQ value. In general, microplastic concentrations decreased with depth below the core surface. Microplastics were detected in 39 of 42 sediment samples in seafloor cores and 19 of 25 samples in marsh cores. In seafloor cores, the earliest detection (above LOD) of microplastics in the sediment was found at Site 7 in 1959, with a concentration of 63 MP particles kg^−1^ DW (30.5 cm depth; Fig. 4). However, only two seafloor cores were long enough to account for accumulation prior to 1960. In marsh cores, microplastic particles were detected at 29 cm, corresponding to 1942, with a concentration of 110 MP particles kg^−1^ DW (Fig. 4). The detectable MP for seabed concentrations ranged from 30 ± 2 to 11,000 ± 750 MP particles kg^−1^ DW, an average of 3300 particles kg^−1^ DW. Marsh core MP concentrations ranged from 42 ± 3 to 31,000 ± 2100 particles kg^−1^ DW with an average of 3500 particles kg^−1^ DW. All cores exhibited a statistically significant exponential increase in microplastic concentrations with time (i.e., up-core, *p* < 0.05), except for Site 5, which had a small sample size (*p* = 0.08).

The color and morphology (i.e., film, fiber, foam, fragment, bead) of all counted particles showed considerable change with time (stratigraphic depth) (Supplemental Figs. 11–14). When considering all samples across seafloor and marsh sites, fibers (50%) and fragments (39%) dominated the MP morphologies, while beads (2%) and foams (3%) were the least commonly observed. Across all sediment cores, color richness ranged from 4 to 12 different color categories per sample, with Shannon diversity (H′) values spanning 0.82 to 1.96. Color diversity increased toward surface layers at several sites, most notably at Site M2, where H′ increased from 1.1 in sediments dated to the 1950 s to 1.9 in surface sediments. Regression analyses showed positive and, in some cases, statistically supported temporal trends, including a significant increase at Site M2 (slope = 0.022 year⁻^1^, *p* = 0.009) and a marginal increase at Site 7 (slope = 0.0166 year⁻^1^, *p* = 0.058). Other sites exhibited stable or weak patterns (e.g., Site 2: slope = 0.0027 year⁻^1^, *p* = 0.43). Particle morphology Shannon diversity ranged from 0.65 to 1.54 across sites. Morphology diversity increased over time modestly at several sites, including Site 7 (slope = 0.018 year⁻^1^, *p* = 0.020), whereas Sites 3 and 5 showed no measurable trend (*p* > 0.5).

The richness in particle morphology and color (i.e., number of different MP morphologies or colors present in a sample) typically increased with time, with samples prior to the 1980 s having the lowest richness values. Before 1960, mostly fibers were observed, with limited coloration. The prevalence of films, foams, and beads markedly increased vertically in the cores, especially after 1980. Over time, across all cores, the proportion of fibers increased slightly, while the proportion of fragments decreased. The three most common MP colors observed were transparent (24%), blue (18%), and black (15%). The least common colors were gold (0.5%), purple (2%), brown (2%), and orange (3%). Morphology and color of MPs varied between sites. Distal sites were dominated by fragments, while proximal sites, the marsh sites, and the offshore site were all dominated by fibers. Particle richness in terms of colors was highest at proximal and marsh sites, and increased through time from past to present.

**Fig. 5:**
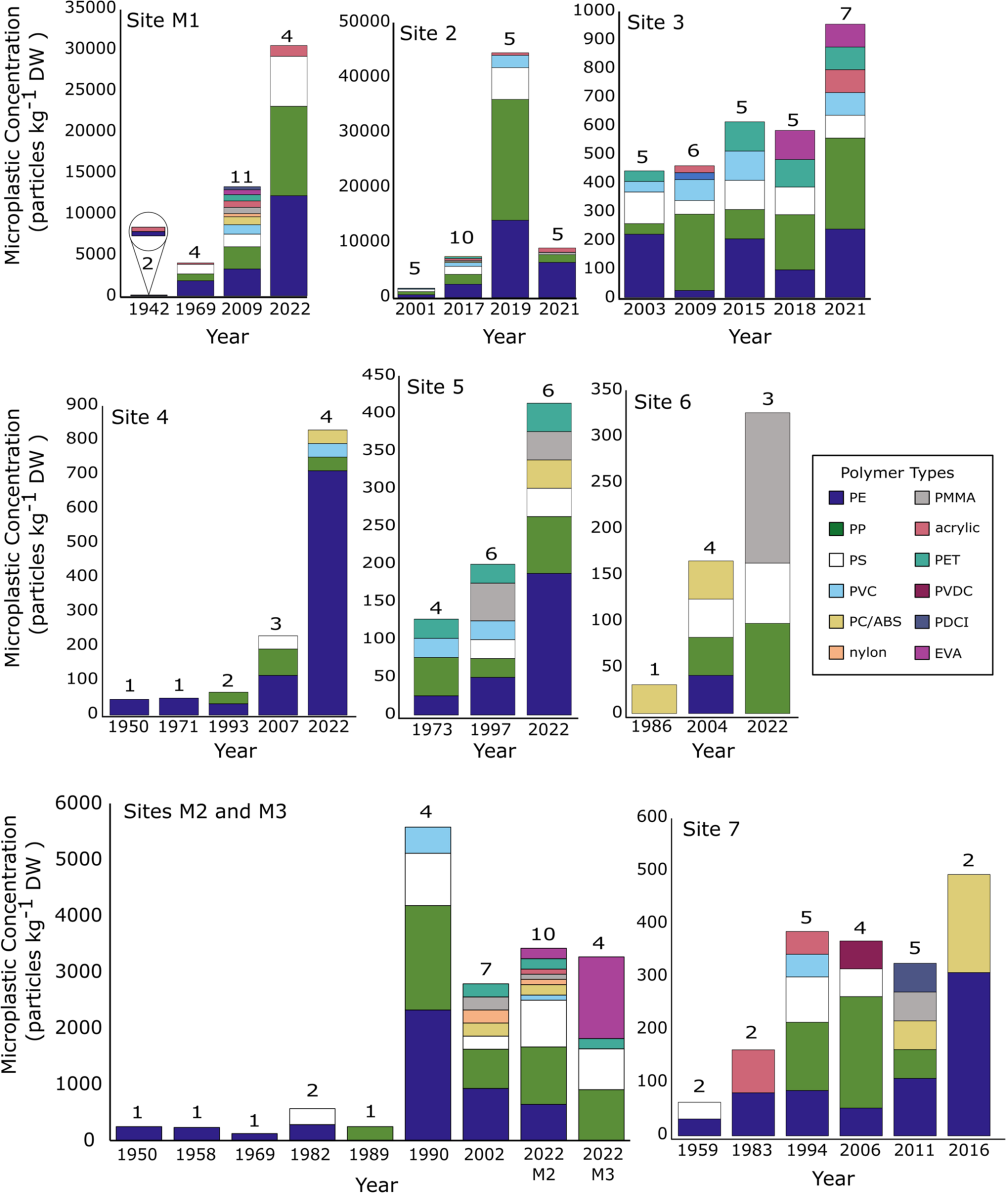
Anthropogenic polymer types identified through time at each site. Twelve different polymers (different colors) were found across the cores, with polymer diversity increasing over time. The number above each bar denotes the number of different plastic polymer types identified in each sample. Polymer type abbreviations are as follows: Polyethylene (PE); polypropylene (PP); polystyrene (PS); polyvinyl chloride (PVC); polycarbonate/acrylonitrile butadiene styrene (PC/ABS); nylon; polymethyl methacrylate (PMMA); acrylic; polyethylene terephthalate (PET); polyvinylidene chloride (PVDC); poly(dicycloundecyl itaconate) (PDCI); ethylene–vinyl acetate (EVA)

MP polymer composition differed between sites and through time. Polymer Shannon diversity ranged from 0.90 to 2.05, with several sites showing upward trajectories consistent with diversification of plastic production (Supplemental Figs. 12–14). Polyethylene (PE; 36.1%), polypropylene (PP; 32.2%), and polystyrene (PS; 11.6%) were the top three polymer types observed across all sites (Fig. 5). Ethyl vinyl acetate (EVA; 0.1%), poly(dicycloundecyl itaconate) (PDCI; 0.7%), and polyvinylidene chloride (PVDC; 1%) were the least common polymer types measured. Consistent with the morphology and color changes, the number of polymer types typically increased through time. During the 1940 s and 1950 s, only PE and acrylic were observed. After 1960, both PP and PS were measured. In the 1970 s, polyethylene terephthalate (PET) and polyvinyl chloride (PVC) were first detected, and polycarbonate/acrylonitrile butadiene styrene (PC/ABS) appeared in the 1980s. Following the 1980 s, the diversity of types increased rapidly, to up to 12 polymers detected overall by 2022. These data mimic the boom in the plastic economy, from less than 10 polymer classes being manufactured in the 1940 s compared to the estimated 300 to 400 distinct polymer classes manufactured in the present day (Andrady and Neal 2009; Mossman 1997). The samples with the most diverse polymer types were a core section from marsh site M1, near Providence, RI dated to be 2009, which contained 11 different polymer types, and 2017 and 2022 samples from Site 2 and marsh Site M2, respectively, which both contained 10 different polymer types. The Narragansett Bay sediment cores showed diversification of polymers through time as might be expected due to increased plastic production and diversification.

**Fig. 6:**
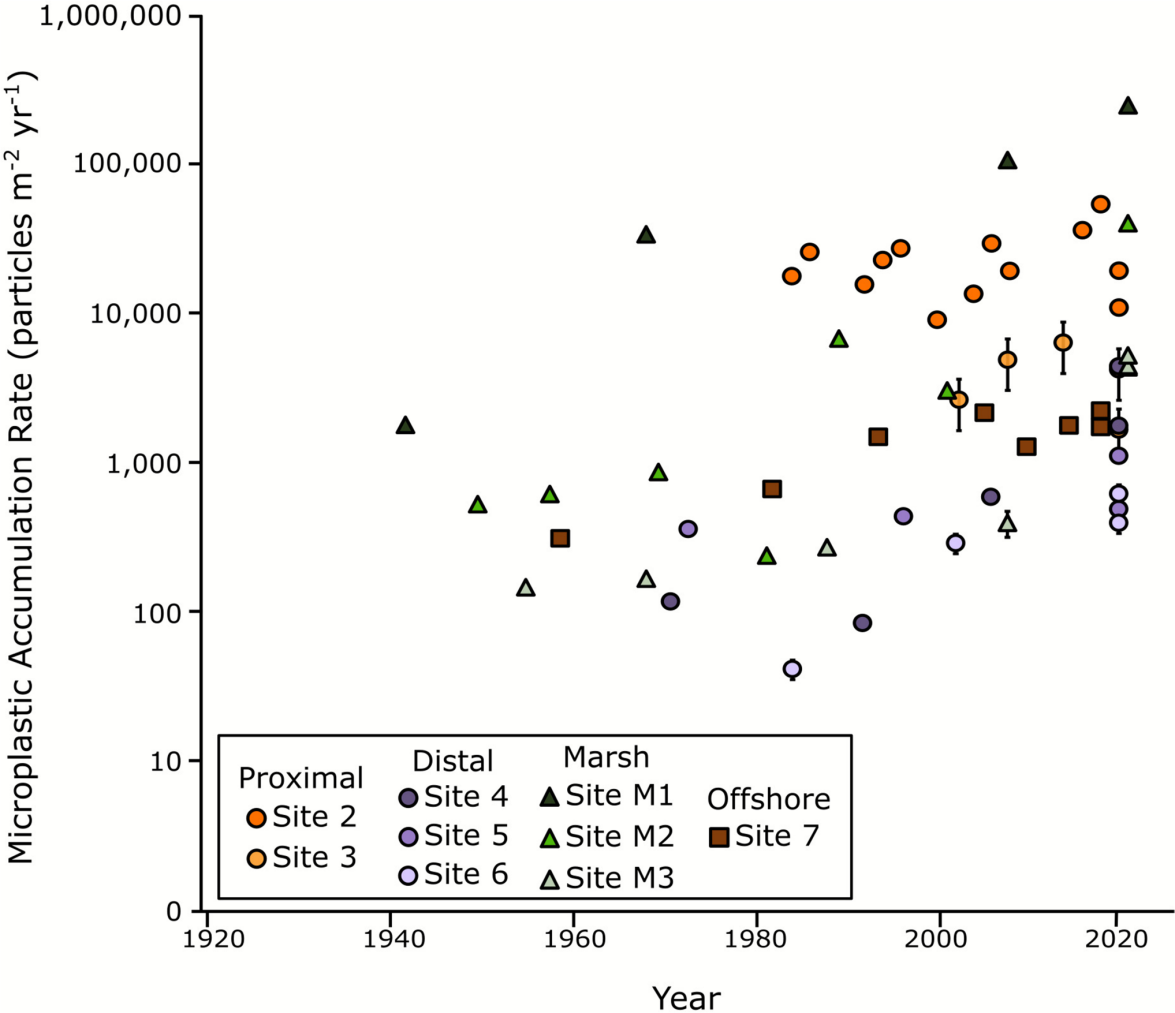
Microplastic accumulation rates (MPARs) increase over time at all sites. Proximal sites (orange circles) and marsh sites (green triangles) accumulate MPs at higher rates than Distal (purple) and offshore (brown) sites. Error bars represent standard error and in most cases are contained within the data point symbol

Microplastic accumulation rates (MPAR; MP particles m^−2^ year^−1^) were calculated using the sediment MARs and down-core concentrations of microplastics at each interval. All sites exhibited increasing MPARs through time, with the marsh sites having the highest rates (> 10,000 particles m^−2^ year^−1^), followed by the Proximal Zone sites (Sites 2–4), offshore Site 7, and Distal Zone sites (Sites 5 and 6; Fig. 6). Marsh sites M1 and M2 consistently accumulated plastics at higher rates than the nearest seafloor site. The highest MPARs, unsurprisingly, were found in the most recently deposited sediment layer representing present-day accumulation and were located at sites closest to Providence and the head of Narragansett Bay for both seafloor and marsh cores. The highest marsh MPAR, 250,000 ± 57,000 MP particles m^−2^ year^−1^ at Site M1, was nearly six times higher than the highest accumulation rate at a seafloor site (44,000 ± 2800 MP particles m^−2^ year^−1^ at Site 2).

## Discussion

### Source influence on and impact of microplastic abundance and particle diversity

This study found exponential increases in microplastic concentrations in ten sediment cores from Narragansett Bay and the Rhode Island Sound, with quantifiable MP contamination dating back as far as 1969. Several other studies worldwide have found microplastics in sediment cores in ocean basins (Brandon et al. 2019), estuaries (Belivermiş et al. 2021; Matsuguma et al. 2017; Uddin et al. 2021; Willis et al. 2017), river basins (Almas et al. 2022), and the Mediterranean Sea (Simon-Sánchez et al. 2022). While the lower limit for the size classification for microplastics varies between studies, from 11 µm (Simon-Sánchez et al. 2022) to 315 µm (Matsuguma et al. 2017), all studies have found an increase in microplastic pollution from the past to present day, and down-core concentrations varied widely due to differences in local conditions. Variances in methodology and size ranges make direct comparisons between studies difficult. Nevertheless, the down-core concentrations of microplastics in Narragansett Bay are comparable to those in other coastal environments.

Narragansett Bay seafloor cores had an average microplastic concentration of 3300 particles kg^−1^ DW based on all samples analyzed, and this is heavily influenced by Sites 1 and 2, which are located just outside the city of Providence. Also, these sites represent only relatively recent sediment accumulation (1984 onward) and have the highest MP concentrations. When considering only seabed cores below this zone, MP concentrations range from 31 to 2000 particles kg^−1^ DW (average 450 particles kg^−1^ DW) (Fig. 4). Based on these data, high concentrations are most prevalent near urban centers where potential MP source points exist. When compared to other studies using sediment cores to estimate MP concentrations through time, Narragansett Bay concentrations are most similar to those found in Tokyo Bay (Matsuguma et al. 2017) and the Mediterranean Sea (Simon-Sánchez et al. 2022) (Table 1). While the ranges of MP concentrations through time vary geographically, it is clear that MP pollution has been a worldwide problem for many decades and that microplastics are now a diagnostic part of the global sediment record.

**Table 1:** Microplastic concentration (MP kg^−1^ dry weight) in sediment core samples from coastal environments

Location	Age range	MP concentration (MP kg^−1^ DW)	Study
Narragansett Bay, RI	1942–2022	31–2000	This study
Tokyo Bay	1952–2001	220–5700	Matsuguma et al. (2017)
Gulf of Thailand	1950–2004	100–330	Matsuguma et al. (2017)
Kuwait Bay	1951–2009	100–500	Uddin et al. (2021)
Mediterranean Sea	1965–2020	706–6939	Simon-Sánchez et al. (2022)

In the Narragansett Bay cores, microplastic composition (i.e., morphology, color, polymer type) varied spatially (Fig. 5; Supplemental Fig. 14). Sites located in the Proximal Zone, in salt marshes, and offshore were dominated by fibers, while Distal Zone sites from the city were dominated by fragments. This variation is mirrored in other studies where more urban-influenced areas with high populations are dominated by fibers (Almas et al. 2022; Uddin et al. 2021; Willis et al. 2017). The predominance of one particle morphology over another, as well as the diversity of colored particles observed, could be influenced by a combination of extraction and analysis methods used, as well as local conditions and point sources, such as the prevalence of wastewater treatment facilities, textile manufacturing plants, stormwater runoff, and industrial inputs. Particle morphology and color are thought to affect the behavior and ecological effects of MPs. For example, the ability to adsorb pollutants differs with morphology due to relationships with size and surface area (Bagheri et al. 2020; Chae and An 2017; Paul-Pont et al. 2018). Previous studies have also found that color influences ingestion risks to organisms, as blue, green, white, and transparent particles are the most likely to be mistaken for a natural food source (Boerger et al. 2010; Fu et al. 2020).

Diversity in color, morphology, and polymer type varied between sites, with more unique combinations of particle types found in urban areas (Proximal Zone) than in more suburban areas (Distal Zone). This indicates that local land use (e.g., urbanization) and activities (e.g., manufacturing, commerce, transportation, marine industries, recreation) likely play an important role in controlling MP particle richness and diversity (Fang et al. 2021). The Proximal Zone and specifically the upper layers of Site 5, just seaward of the port of Quonset, contained more polymer types than other sites, reflecting the diversity of plastic pollution sources near urban centers and industrial areas. Change in particle morphology and polymer type from the urban center and toward the open ocean is a pattern reflected in other sedimentary studies, suggesting that distance from MP sources has a strong influence on particle diversity (Fulfer and Walsh 2023; T. Wang et al. 2019; Zhao et al. 2015). However, research on plastics in ocean gyres supports great diversity making it seaward, and therefore refined models of microplastic transport and deposition will be needed to better understand source to sink dynamics (Courtene-Jones et al. 2022; Zhao et al. 2015).

The benthic environment is an important feeding environment for many coastal species (Anderson and Lovvorn 2008; Gittman and Keller 2013). Previous studies, although disparate in scope and scale, have indicated the presence of MP particles in the tissues of benthic organisms in estuarine and coastal environments, and this is a concerning finding considering the role these organisms play in the ecosystem and, in many cases, as food sources for coastal communities (Baechler et al. 2020b, a; Baechler et al. 2020b, a; Courtene-Jones et al. 2022; Davidson and Dudas 2016; C. Fang et al. 2018; Li et al. 2015; Mathalon and Hill 2014; Setälä et al. 2016; Su et al. 2020; Van Cauwenberghe et al. 2015). Laboratory-based ecotoxicology studies have revealed ingestion by shellfish species that leads to a number of deleterious effects, such as reduced growth rates and energy uptake, increased stress, inflammation, DNA damage, neurotoxicity, mortality, and reduced fecundity (Browne et al. 2008; Green 2016; Horn et al. 2020; Ribeiro et al. 2017; Sussarellu et al. 2016; von Moos et al. 2012; Woods et al. 2018). Ecotoxicology studies are used to determine predicted no-effect concentrations (PNEC), or the limit below which no adverse effects to an ecosystem are measured (Cooper et al. 2008; Dussault et al. 2008). The current accepted PNEC for exposure of benthic biota to microplastic pollution is 540 MP particles kg^−1^ (Everaert et al. 2018; Koelmans et al. 2023; Van Cauwenberghe et al. 2015; Yang et al. 2023), and all but one of our sites has exceeded this concentration in the surface sediment (Supplemental Fig. 15). Proximal marsh and seafloor sites exceeded the PNEC threshold as early as 1968 (marsh site M1) and 1984 (seafloor site 2), respectively. The outer edge of the Distal Zone marsh, site M2, crossed the critical threshold in 1970, while the first seafloor Distal Zone site to exceed 540 MP particles kg^−1^ was Site 4 in 2022. However, this PNEC value is a first-order estimate derived from limited effect data and should therefore be interpreted as an indicative threshold to screen for ecological risk and not a definitive regulatory value. While this study did not examine the impact of MP pollution on the local biota, the high concentrations reported here should serve as a warning for potential negative effects on benthic biota in the future.

Microplastics in Narragansett Bay co-locate with other contaminants, including heavy metals and organic pollutants (Nixon et al. 1986; Latimer and Quinn 1996; Hartmann et al. 2005; Schmidt et al. 2020), raising concerns about combined exposure and enhanced pollutant mobility. The high surface area and hydrophobicity of many plastic polymers allow MPs to adsorb metals (e.g., Pb, Cd, Hg) and organic contaminants from surrounding waters and sediment, sometimes at concentrations orders of magnitude higher than ambient sediments (Holmes et al. 2012; Rochman et al. 2013a, b). Estuaries further amplify this risk because fine, organic-rich, low-energy sediments enhance both microplastic deposition and contaminant binding, promoting the formation of microplastic–contaminant complexes that can persist and accumulate over time (Li et al. 2018; Richard et al. 2019). Pharmaceuticals and personal-care products can also sorb to microplastics or biofilms on their surfaces, creating additional pathways for trophic transfer and potential ecological toxicity (F. Wang et al. 2020). The co-occurrence of microplastics and chemical contaminants in Narragansett Bay therefore may represent a multi-stress exposure scenario with implications for benthic organisms, biogeochemical cycling, and long-term contaminant storage or remobilization (Turner and Holmes 2015).

### Sedimentation and microplastic sequestration

Quantification of down-core microplastic concentrations and the rates of MP accumulation at different sites sheds light on historical patterns of microplastic accumulation, controls on rates of sedimentation, and potential changes in MP sources over time. In addition, the data presented here contribute to the body of knowledge on the long-term sequestration of MPs and the potential removal of MPs from the coastal environment through burial. Before discussing MP accumulation, it must be recognized that coastal and marine river–derived sediment accumulation is governed by an array of factors (Walsh and Nittrouer 2009), and more specifically, the sedimentation in estuaries is influenced by sediment input, morphology, tides, currents, and more (Dalrymple et al. 2012; Dyer 1995; Prandle 2009). Microplastics concentrations and accumulation are necessarily going to be affected by these aspects, and future studies should strive to evaluate the role of sedimentary dilution and transport processes. A fundamental concept for sedimentation is that sufficient water depth, i.e., “accommodation space” in stratigraphy (Jervey 1988; Muto and Steel 2000), is needed to limit the transport and/or remobilization of materials being input. Narragansett Bay is a fjord-like estuary with relatively deep water, little sediment supply, minimal tides (McMaster 1960), and locally weak (< 5 cm/s) subtidal current (Pfeiffer-Herbert et al. 2015), and given these factors, it is conducive to trapping sediment.

Proximal Zone sites in Narragansett Bay have higher sediment accumulation rates as well as higher MP concentrations, and thus greater MP accumulation rates, relative to Distal Zone sites. This situation in the Bay points to both increased ability to accumulate sediment in the Proximal Zone due to available accommodation space as well as higher inputs of plastic. Other estuaries may not have the space to accumulate sediment or the elevated concentrations of microplastics, and it is likely that the sources of sediments and microplastics in many systems will not be co-located. Also, it is expected that river sediment fluxes may dilute rather than enhance microplastic accumulation rates in some systems; the Narragansett Bay has minimal sediment influx due to its watershed geology, human history, and damming.

MP accumulation rates (MPARs) change with time, potentially due to varying MP concentrations or changes in sediment accumulation. This study assumed constant sediment accumulation based on steady-state ^210^Pb profiles below the mixed layer and, as a result, the vertical changes in MPARs are driven by MP concentrations. MPARs in Bay sites ranged from 41 ± 6 to 44,000 ± 2800 MP particles m^−2^ year^−1^, with an average accumulation rate of 10,000 MP particles m^−2^ year^−1^; however, these rates vary in space and time (Fig. 6). In the Proximal Zone, MPARs vary from 83 ± 6 to 44,000 ± 27800 MP particles m^−2^ year^−1^, and in the Distal Zone, the rates are much lower, between 41 ± 6 and 2200 ± 180 MP particles m^−2^ year^−1^. MPARs increased exponentially through time in all sampled zones (Fig. 6), with this signal of plastic sedimentation propagating down the Bay through time, and this is analogous to the delay and modification of other sedimentary signals in dispersal systems (Romans et al. 2016). This is the first North American study to document a plastic signal propagation along a marine sedimentary system. Other studies have also shown varying MPARs; for example, Simon-Sánchez et al. (2022) analyzed a core with a constant sediment accumulation rate from 104 m water depth in the Mediterranean Sea and found the MP (> 11 µm) burial rate increased from 865 MP particles m^−2^ year^−1^ in 1973 to 8507 MP particles m^−2^ year^−1^ in 2016. Site 7, at 57 m water depth, is the most comparable core in this study, and MPAR increased from 770 MP particles m^−2^ year^−1^ in 1973 to 1800 MP particles m^−2^ year^−1^ in 2016. In contrast, at Site 2, MPAR increased from 15,000 to 25,000 MP particles m^−2^ year^−1^ in the Proximal Zone and at Site 4, MPAR increased from 57 to 1000 MP particles m^−2^ year^−1^ in the Distal Zone across the same timeframe.

Inventories of accumulated contaminants can be calculated for whole cores or for discrete sediment age timeframes using accumulation rates and contaminant concentrations. At the Mediterranean Sea site, the accumulated MP inventory since 1965 was 1.44 × 10^6^ MP particles m^−2^ (Simon-Sánchez et al. 2022). Accumulated MP inventories since 1965 in Narragansett Bay ranged from 1.0 × 10^4^ MP particles m^−2^ at Site 6 to 1.0 × 10^6^ MP particles m^−2^ at Site 2. Marsh sites had even higher accumulated inventories, as high as 5.2 × 10^6^ MP particles m^−2^ at Site M1, with an average of 1.9 × 10^6^ particles m^−2^ for all marshes measured. Accumulation of organic matter and contaminants often increases with decreasing sediment grain size (Bergamaschi et al. 1997; Chakraborty et al. 2015) but, as in other MP studies, this relationship was not observed for MPs in Narragansett Bay sediment (Alomar et al. 2016; Browne et al. 2010; Van Cauwenberghe et al. 2015).

**Fig. 7:**
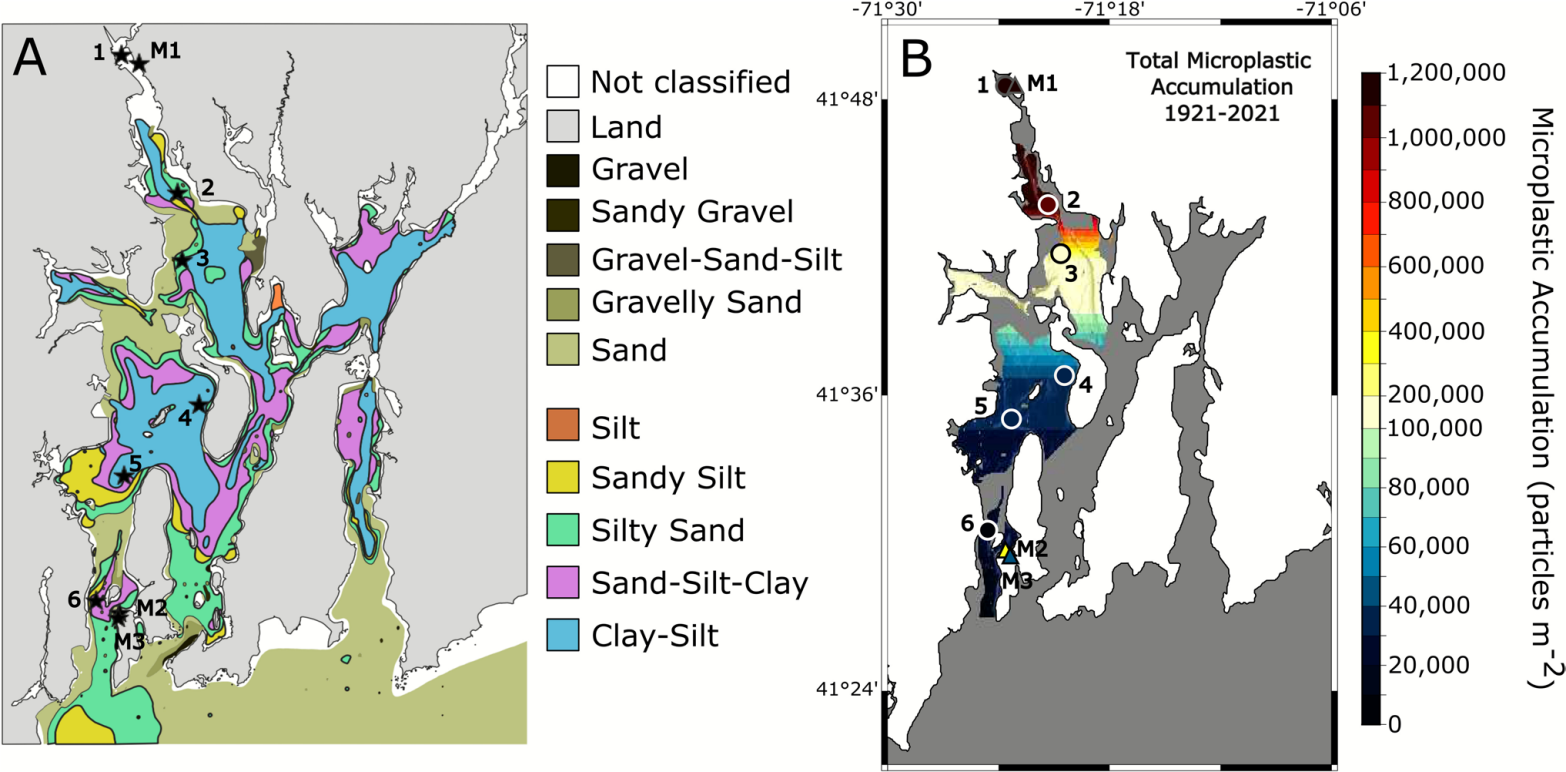
Microplastic accumulation rates in Narragansett Bay increased through time, with the earliest detection occurring in 1942. Microplastic samples were taken in deep basins containing finer grain sediments. Modified from McMaster (1960), the sediment grain size distributions in Narragansett Bay (**A**) were used to determine areas comparable to sampling zones (McMaster 1960). Areas characterized by finer grained sediments (colored zones with black outlines) were included in a model of MP accumulation over the past 100 years (**B**). Microplastic accumulation over the past 100 years is highest in the Proximal Zone (warm colors) and lowest in the Distal Zone (cool colors)

Estimates of MP storage in this system are based on some assumptions and limited data, and thus should be treated as a preliminary estimate in need of further investigation. As noted, cores were only collected in mud-dominated areas where fine sediment is accumulating. Therefore, storage of MP for the West Passage of the Bay was only calculated for the mapped areas of fine-grained sediment (McMaster 1960) (Fig. 7). Using linear relationships between distance from the urban center (latitude) and MP accumulation rates in the Proximal and Distal Zones, estimates of MP accumulation for each decade and over the past century were calculated (see Supplemental Methods; Supplemental Fig. 16). Based on only areas of fine sediment accumulation, the average MP storage decreases down the Narragansett Bay, but increases with time, from 4.5 × 10^11^ particles year^−1^ in the 1970 s to 6.5 × 10^11^ particles year^−1^ in the 2010 s (Supplemental Fig. 17). Over the past century (1921–2021), the sediments in those same fine sediment basins have accumulated 1.9 × 10^13^ MP particles (18.7 trillion MPs) (Fig. 7). When considering the entirety of the West Passage, this number rises to 38 trillion particles. Using a conservative MP particle mass estimate of 58.6 µg per particle (Fulfer and Walsh 2023), this amounts to 2300 tonnes of microplastic pollution. While the West Passage only accounts for about 24% of the total Narragansett Bay area, it includes the Lower Providence River, which this study and Fulfer and Walsh (2023) show holds the most MP pollution. Other studies have found that MP properties (e.g., size variability; carbonyl index variability) did not vary with depth in sediment cores, suggesting that MPs are not appreciably degraded and thus largely preserved beneath the mixed layer (Corcoran et al. 2015; Simon-Sánchez et al. 2022). The limited studies in other systems, along with the high levels of MP sequestration measured throughout Narragansett Bay, suggest that estuaries, where accommodation allows, may serve as important long-term MP pollution sequestration areas.

The sediments of Narragansett Bay have long been viewed as sequestration environments for anthropogenic pollutants, including heavy metals (Goldberg et al. 1977; Nixon et al. 1986; Schmidt et al. 2020), and a few earlier studies have reported down-system trends for heavy metals and organic pollutants (Hartmann et al. 2005; Latimer and Quinn 1996; Santschi et al. 1984). Nixon et al. (1986) attempted budgets by comparing the total inputs to the system to the storage of nutrients, heavy metals, and petroleum hydrocarbons in the sediment of Narragansett Bay. Heavy metals (Mn + Cd + Pb + Cu) and petroleum hydrocarbons accumulated in Narragansett Bay sediments at rates up to 0.77 g m^−2^ year^−1^ and 3.8 g m^−2^ year^−1^, respectively (Nixon et al. 1986; Nixon and Fulweiler 2012). MP pollution was relatively low in the 1980 s, accumulating between 0.002 (Site 6) and 2.0 (Site 2) g m^−2^ year^−1^ and has rapidly increased since then. Approximately 70 to 100% of Cu (70–96%), Pb (80–100%), and petroleum hydrocarbons (25–75%) inputs are estimated to be stored long-term in the sediment (Nixon et al. 1986; Nixon and Fulweiler 2012). A MP budget is not attempted here as inputs of MPs over time are poorly constrained but, for comparison, 70 to 99% of microplastics input to the marine environment are believed to be deposited on shorelines and in marine sediments (Frias et al. 2016; Law 2017). While mitigation efforts have reduced the input and accumulation of heavy metals and hydrocarbons in Narragansett Bay sediments (Narragansett Bay Commission 2018; RI dem Office of Water Resources 2016), the lack of plastic pollution mitigation has allowed MP accumulation to increase rapidly. The success of mitigation strategies in the past should provide a framework and offer hope for the possibility of ameliorating this system through directed action on plastic pollution.

### Marshes serve as efficient microplastic traps

Bricker (1993) found salt marsh cores in the Proximal Zone of Narragansett Bay were more enriched in Pb, Cu, and Zn, reflecting the proximity to sources at the head of the estuary. The same trend is seen for microplastics between Site M1 at the head of the estuary and sites M2 and M3 near the mouth. Microplastic accumulation in the marsh sites of Narragansett Bay far outweighed the concentrations found in nearby bay sites (Fig. 4). In the Proximal Zone, Site M1 trapped plastics at concentrations two to three times higher than Site 1, with concentrations between 2000 and present day ranging from 13,000 ± 890 to 31,000 ± 2100 particles kg^−1^ DW. Site 1 MP concentrations inferred over the same assumed time period, based on the fact that the most recent dredging occurred in 2004 (USACE and INSPIRE Environmental 2020), ranged from 5100 ± 340 to 11,000 ± 750 particles kg^−1^ DW. In the Distal Zone, the outer marsh at Fox Hill (Site M2) had higher MP accumulation than the inner marsh (Site M3), but both M2 and M3 still far outpaced the nearby seabed, Site 6. From 1980 to present day, Site 6 MP concentration ranged from 31 ± 2 to 330 ± 22 particles kg^−1^ DW. For the same time range, the inner marsh (M2) MP concentrations were over tenfold higher, and the outer marsh (M2) had MP concentrations fifty times higher than Site 6. MPARs for the past century were over four times higher in marsh sites (4.8 × 10^4^–56.4 × 10^5^ MP particles m^−2^) than Bay sites (1.2 × 10^4^–12 × 10^5^ MP particles m^−2^; Fig. 6).

Salt marshes act as efficient traps for microplastics and other contaminants when compared to bare or non-vegetated seafloor due to a combination of hydrodynamics, plant structure, and sediment properties. Vegetation, including stems and leaves, reduces current velocities and turbulence, allowing particles to settle (Kretz et al. 2021; Xu et al. 2022). The roots of salt marsh plants can act as a sieve, filtering and capturing fibers and fragments as water rises during high tide (McIlwraith et al. 2024; Stead et al. 2020). Salt marshes typically consist of fine-grained, organic-rich sediments, which are naturally cohesive and encourage flocculation of MPs with fine-grain sediment, enhancing MP deposition and accumulation when compared to sandier sediment (Paduani 2020). Finally, higher sediment accretion rates at salt marshes, along with bioturbation by active sediment biota, may increase burial of MPs in salt marsh sediment (Ogbuagu et al. 2022).

The efficient trapping of microplastics in salt marsh environments has been seen in other recent studies (Almeida et al. 2023; Egea et al. 2023; Pinheiro et al. 2022; Yao et al. 2019). Stead et al. (2020) found a 66% decrease in microfiber abundance in salt marsh adjacent waters between flood and ebb tides, indicating trapping of two-thirds of the particles in the intertidal zone. Ogbuagu et al. (2022) measured negligible erosion of microplastic particles from salt marsh sediments during tidal flow velocities (0.51 m s^−1^) but consistent erosion of microplastics from mudflats at velocities greater than 0.26 m s^−1^, indicating the positive impact of vegetation on the MP trapping efficiency of salt marsh environments. Evidence indicates that both macro- (not studied here) and microplastics are being trapped in salt marsh environments (Li et al. 2020; Weinstein et al. 2016), with the degradation of macroplastics into microplastics in as little as 8 weeks, leading to additional concerns about intertidal marshes serving as both sources and sinks of MPs, particularly during high velocity and storm conditions (Weinstein et al. 2016).

Lloret et al. (2021) found that salt marsh sediments effectively capture microplastic particles, with accumulation positively correlated with the local level of urbanization. For salt marshes in New Bedford, MA, urban development currently accounts for more than 50% of the land, and this has resulted in over an order of magnitude increase in MP abundances in the sediment (Lloret et al. 2021). Site M1, a marsh in the Proximal Zone, has 30% more urbanized, or impervious, land surrounding it and 3 to ×10 the level of microplastic contamination in the upper sediment layers when compared to M2 and M3 in the Distal Zone. Like marshes, seafloor sites also suggest a land-use connection. Proximal seafloor sites (Sites 1, 2, and 3) are surrounded by 17–39% more impervious land than Distal sites and have 3 to ×55 the level of MP contamination. This evidence further reinforces that urban areas and impervious surfaces increase the input of MPs to nearby wetlands and water bodies, likely reflecting many additional sources (Bell et al. 2019; Hanh Nguyen et al. 2023).

### Impacts of dredged material disposal on the microplastic sedimentary record

Maintenance of intracoastal waterways, channels, and ports often requires sediment to be regularly dredged from the waterway and then deposited at either a land or aquatic based offsite disposal site (Myszewski 2015; Windom and Stickney 1976). Recent efforts have begun to assess the contaminant-related impacts associated with microplastics in freshwater and marine dredged waterways (Constant et al. 2021; Ji et al. 2021; Wilkens et al. 2020). Site 1 is located in a dredged portion of the Lower Providence River, and the core had varying ^210^Pb activity versus depth, reflective of non-steady-state and likely rapid accumulation (Bentley and Nittrouer 1999; Ryan-Mishkin et al. 2009), precluding the construction of an age model (Supplemental Fig. 18). The variable ^210^Pb activity and grain size composition are likely due to this core being in the dredged and frequently disturbed Providence River channel. This channel was dredged in 1971, 1976, and 2004. From 2003 to 2004, six confined aquatic disposal (CAD) cells were created in the Lower Providence River for the disposal of dredge material that was deemed unsuitable for offshore dumping. These CAD cells were intermittently filled between 2005 and the present day (USACE & INSPIRE Environmental 2020). In a 2021 survey, the sediment surface of CAD cell 3R, where Site 1 is located, still sat approximately 2 m below the surrounding channel depth (USACE & INSPIRE Environmental 2020). Based on this, it is hypothesized that the dredging created accommodation space, which then allowed the rapid accumulation of the CAD cell depression where Site 1 is located. This could further explain the consistently high MP concentrations in the upper 40 cm of the core, which averaged 9000 ± 1700 MP particles kg^−1^ DW.

Interestingly, Site 7 was positioned less than 10 km southeast of a heavily used dredge material disposal site. The Rhode Island Sound Disposal Site (RISDS) is an 1800 × 1800 m area centered at 41°13.850′ N, 71°22.817′ W, approximately 16.7 km south of Point Judith, RI (USACE 2021) (Fig. 1). Dumping areas near Site 7 have been utilized since the 1960s. From 2003 to 2020, over 5 million cubic meters of dredged material from the Providence River and other nearby dredging projects was dumped at RISDS (USACE 2021; USACE & INSPIRE Environmental 2020) (Supplemental Fig. 18). Given the high MP contamination in the Providence River, it is postulated that dredged material disposed of offshore may have influenced MP concentrations at Site 7. Plastic particles are typically less dense than sediment particles and may be further transported by local currents from a dump site before accumulating on the seafloor (Stride et al. 2024; He et al. 2021). Dredging of MP contaminated river and coastal sediments poses the risk of remobilization of sedimentary MPs and consequential dispersal into the surrounding terrestrial and aquatic ecosystems. Studies have shown increased MP concentrations in areas surrounding dredge sediment piles, indicating release from the piles into the surrounding environment (Ji et al. 2021; Yi et al. 2023). Dredged sediments may be treated to remove pollutants, but currently, no dredge treatment technologies remove all MP particles due to their diversity in size, density, and chemical makeup (FRTR 2020). While U.S. Army Corps of Engineers (USACE) and the U.S. EPA require studies on the environmental impact of ocean dumping of dredge sediment, there are currently no federal guidelines or restrictions on MP contaminated sediments (U.S. Epa 2015a, b). The Green Book, or Ocean Testing Manual, contains guidelines on ocean dumping, including guidelines for chemical, biological, and physical evaluations of the impact of dredged waters and sediments. According to this document, uncharacterized materials are prohibited from ocean disposal, as are any materials that have proven adverse effects on human health or the marine ecosystem (U.S. Epa 2015a; U.S. EPA,, 40 CFR 227.5(c)). As more research is conducted on the negative consequences of MP pollution on both human health and ecosystems, it may be time to revise the Green Book to consider this new class of pollutants. At any rate, considerations for remobilization of MPs will need to be considered during the dredging, treatment, and disposal processes to assess and minimize the potential environmental impacts.

## Conclusions

In summary, plastic production and use has been increasing exponentially since the 1950 s, and this trend is clearly reflected in the sediment record of Narragansett Bay and likely accumulating coastal environments around the world. Narragansett Bay has, unfortunately, been the subject of numerous historical pollution studies, and despite mitigation efforts for contaminants such as heavy metals and wastewater nutrients, MP pollution has neither been addressed nor mitigated over the past 75 years. This study documents increasing microplastic contamination and increased polymer diversity (morphology, color, and polymer type) from 1942 to the present day, with highest concentrations near urban areas and industrial ports, where permeable surfaces are limited and sources of pollution are concentrated. Remarkably, ecologically important salt marsh habitats in the Proximal and Distal Zones of this estuary are trapping up to ×10 more microplastics than the nearby unvegetated seabed, eliciting concern for the benthic and aquatic biota that rely on these important habitats. While the patterns observed in Narragansett Bay are likely not surprising, the pervasiveness of microplastic pollution throughout space and time should serve as a warning of the permanence of this type of pollution and the urgency with which action must be taken.

## Supplementary Information

Below is the link to the electronic supplementary material.

ESM1 (DOCX 33.8 MB)

ESM2 (XLSX 80.0 KB) 

## Data Availability

All data used in this study are available at the Zenodo open science data repository at 10.5281/zenodo.16914510.
