# Supplementary material for: Exponentially increasing microplastic accumulation in an urban estuary: insights from the Narragansett Bay, Rhode Island seafloor
Source: Environ Sci Pollut Res Int. 2025 Dec 19;32(57):30899–920. doi: 10.1007/s11356-025-37295-2 (PMC12811374; doi:10.1007/s11356-025-37295-2)
Supplement: Supplementary file 1 — (DOCX 33.8 MB) [file 11356_2025_37295_MOESM1_ESM.docx]

**Supplemental Materials**

Exponentially Increasing Microplastic Accumulation in an Urban Estuary: Insights from the Narragansett Bay, Rhode Island Seafloor

Victoria M. Fulfer^1,2^, J. P. Walsh^1^ and D. Reide Corbett^3^

^1^Graduate School of Oceanography, University of Rhode Island, Narragansett, RI, USA

^2^The 5 Gyres Institute, Santa Monica, CA, USA

^3^Coastal Studies Institute, Eastern Carolina University, Wanchese, NC, USA

**1. Supplemental Methods**

**For MP accumulation from 1970 – 1980, the following equations were used:**

For latitudes falling north of the northernmost core in the Proximal zone (latitude >= 41.729), an MP storage of 46,500 particles was assumed, as this was the highest calculated accumulation for a collected core in the Proximal zone in the 1970s.

In the Proximal Zone (latitudes < 41.729 and > 41.690925):

MP Storage = 381,080 x Latitude – 15,885,500

In the Distal Zone (latitudes < 41.690925):

MP Storage = 10,413 x Latitude – 432,390

**For MP accumulation from 2010 – 2020, the following equations were used:**

For latitudes falling north of the northernmost core in the Proximal zone (latitude >= 41.729), an MP storage of 68,800 particles was assumed, as this was the highest calculated accumulation for a collected core in the Proximal zone in the 2010s.

In the Proximal Zone (latitudes < 41.729 and > 41.690925):

MP Storage = 569,820 x Latitude – 23,753,900

In the Distal Zone (latitudes < 41.690925):

MP Storage = 10,370 x Latitude – 430,160

**For MP accumulation from 1920 – 2020:**

For all estimates of MP accumulation since 1920, any core that did not penetrate deep enough to reach an MP concentration of 0 particles kg^-1^ (e.g., Site 2), the deepest (oldest) concentration was extrapolated to reach 0 particles kg^-1^ in 1942, which corresponds to the first detection of MPs in a Narragansett Bay core. For latitudes falling north of the northernmost core in the Proximal zone (latitude >= 41.729), an MP storage of 1,180,700 particles was assumed, as this was the highest calculated accumulation for a collected core in the Proximal zone since the 1920s.

In the Proximal Zone (latitudes < 41.729 and > 41.690925):

MP Storage = 27,059,174.086 x Latitude – 1.1279742442*10^9

In the upper Distal Zone (latitudes < 41.690925 and > 41.60925):

MP Storage = 1,368,462.775 x Latitude – 56,904,939.749

In the lower Distal Zone (latitudes < 41.60925):

MP Storage = 233,770.911 x Latitude – 9,689,372.152

**2. Supplemental Figures**


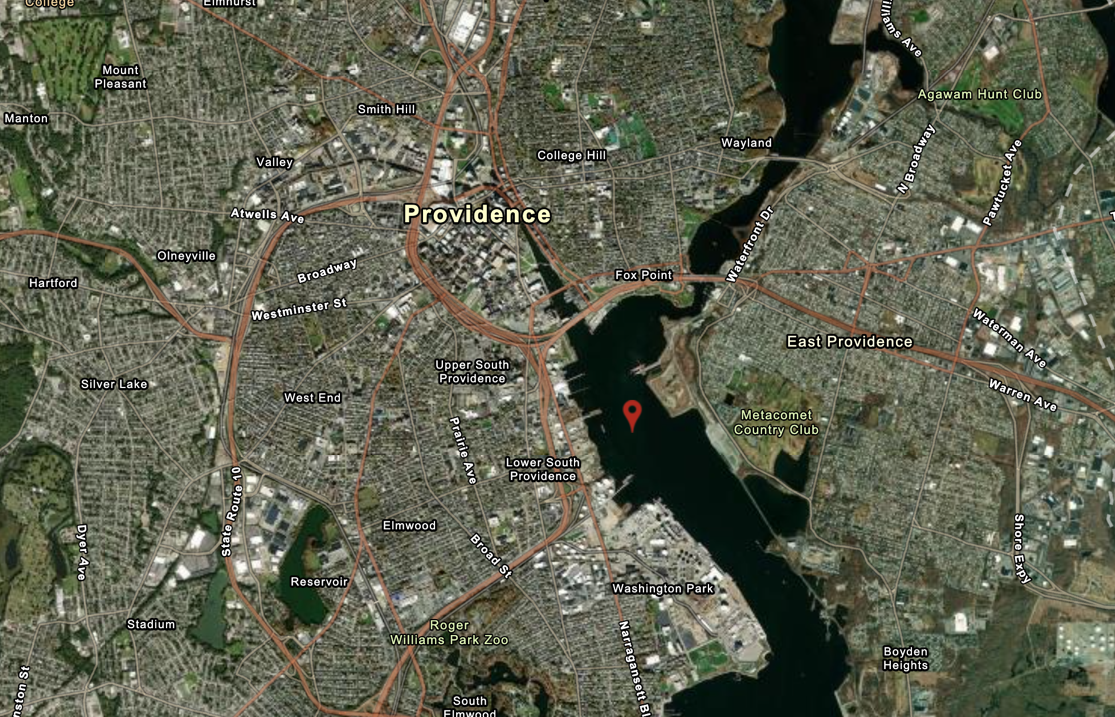


Lower Providence River

Site 1

**Supplemental Figure 1.** Site 1 location in the Lower Providence River.


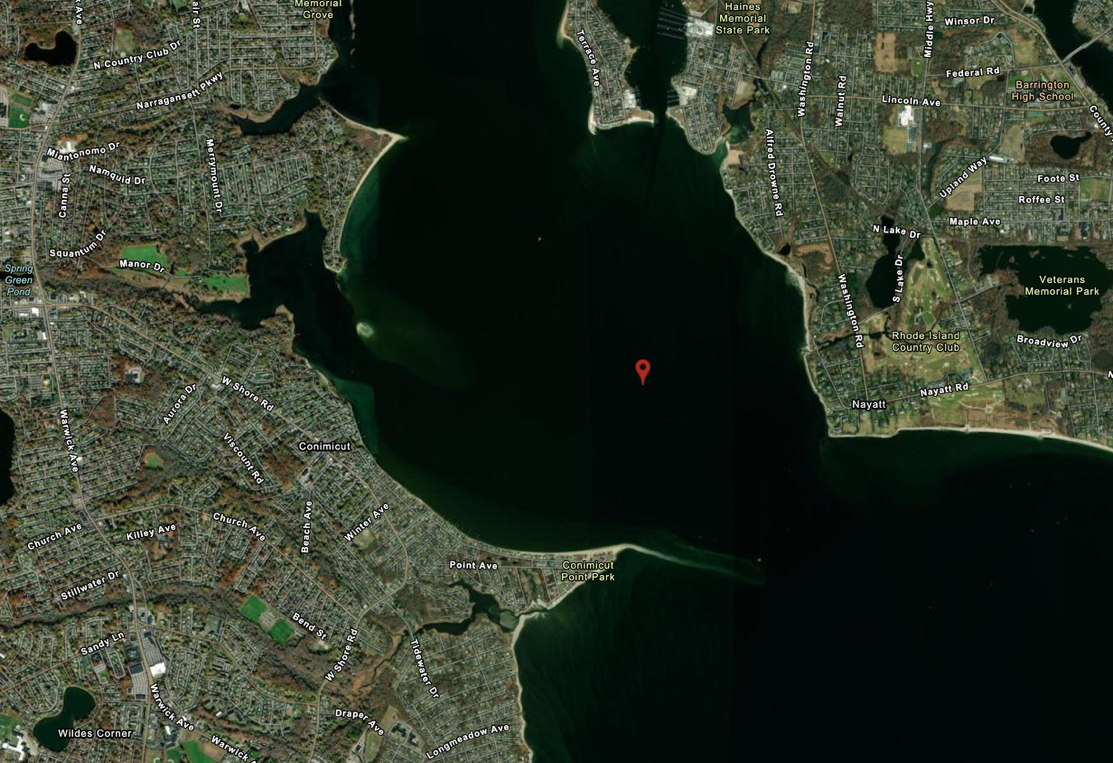


Site 2

Conimicut Point

**Supplemental Figure 2.** Site 2 location offshore Conimicut Point.


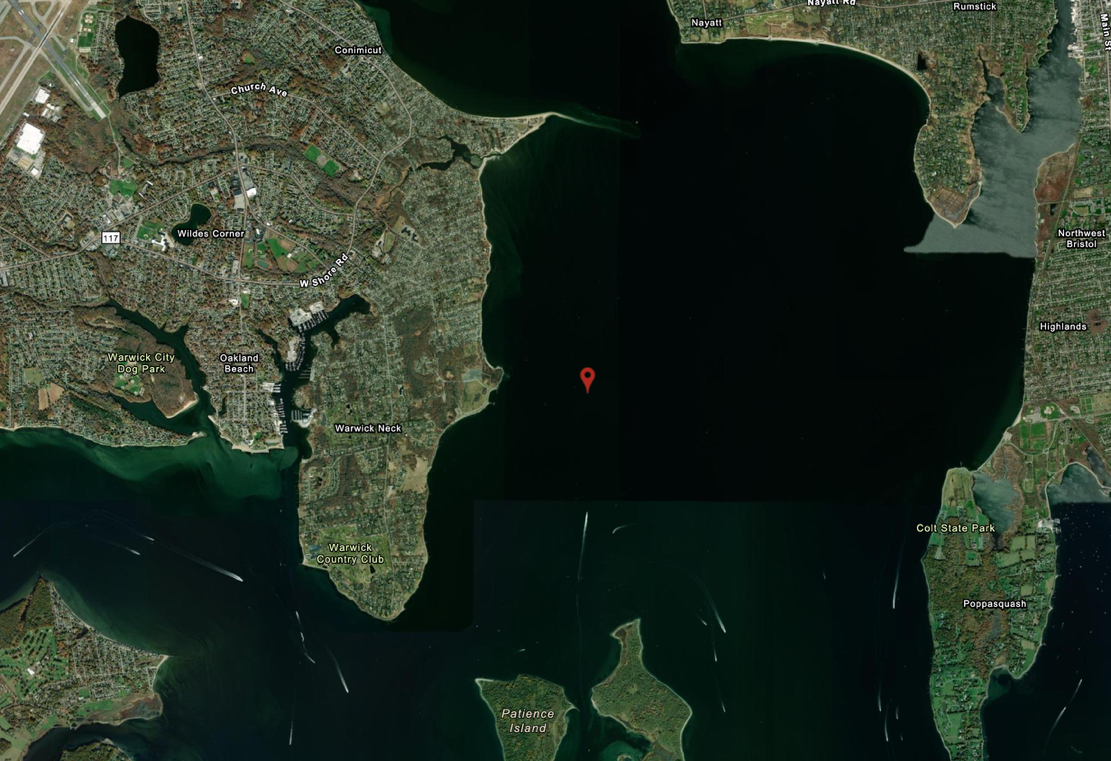


Site 3

Rocky Point Park

**Supplemental Figure 3.** Site 3 location offshore Rocky Point State Park.


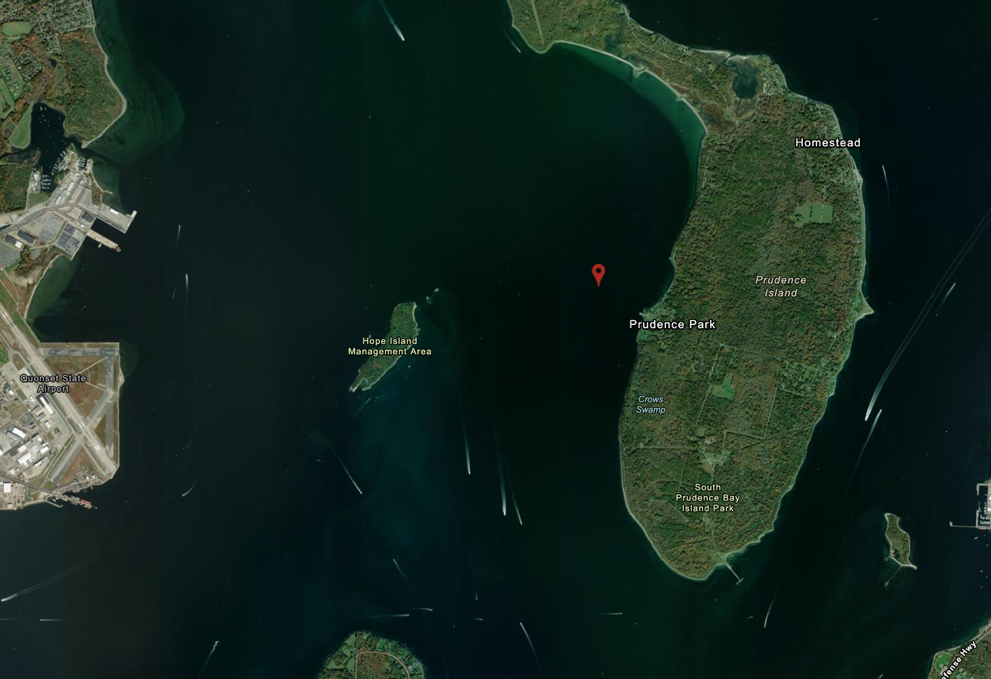


Jenny’s Creek Shellfish Management Area

Site 4

Prudence Island

**Supplemental Figure 4.** Site 4 location off the western shore of Prudence Island. To the east is Quonset Airport. The yellow box indicates the Jenny’s Creek Shellfish Management Area.


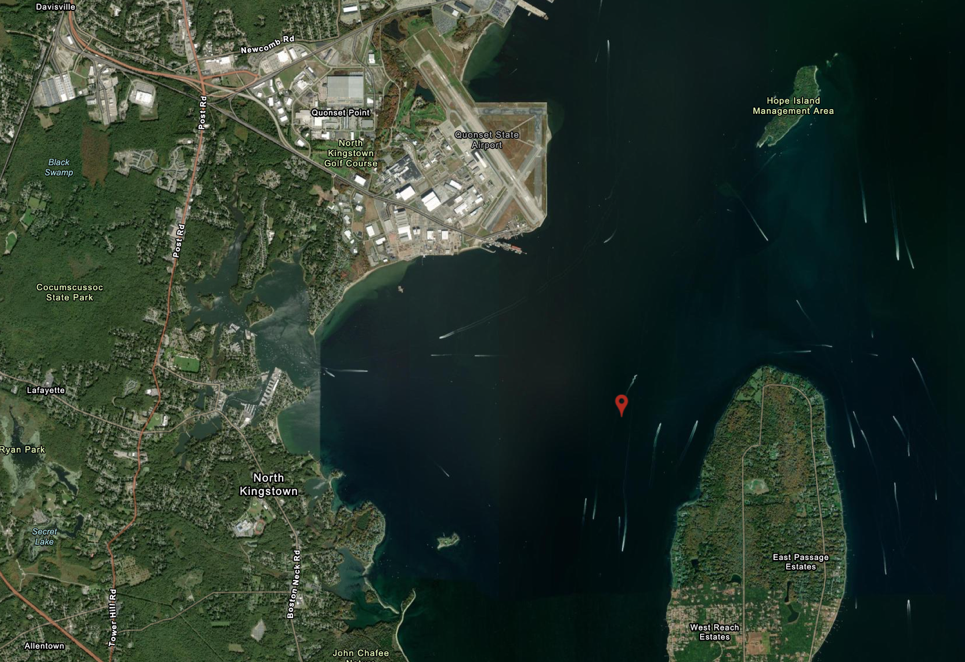


Quonset Airport

Port of Davisville

Site 5

Conanicut Island

**Supplemental Figure 5.** Site 5, located southeast of the Port of Davisville and Quonset Airport.


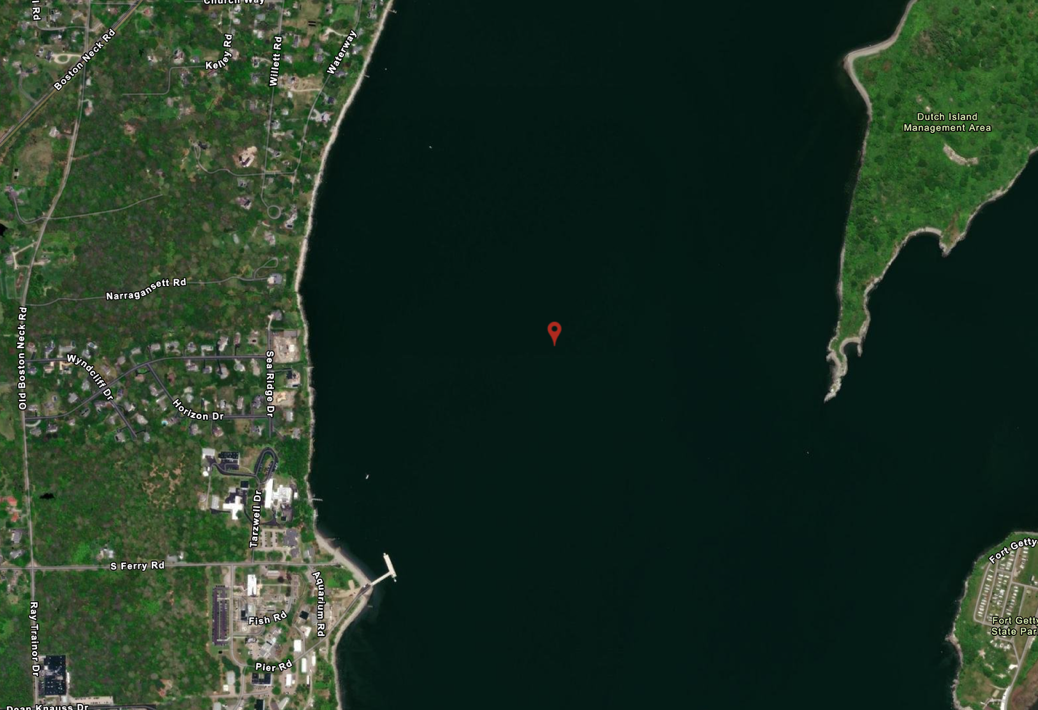


Dutch Island

Site 6

URI Bay Campus

**Supplemental Figure 6.** Site 6, located east of the University of Rhode Island (URI) Bay Campus and west of Dutch Island.


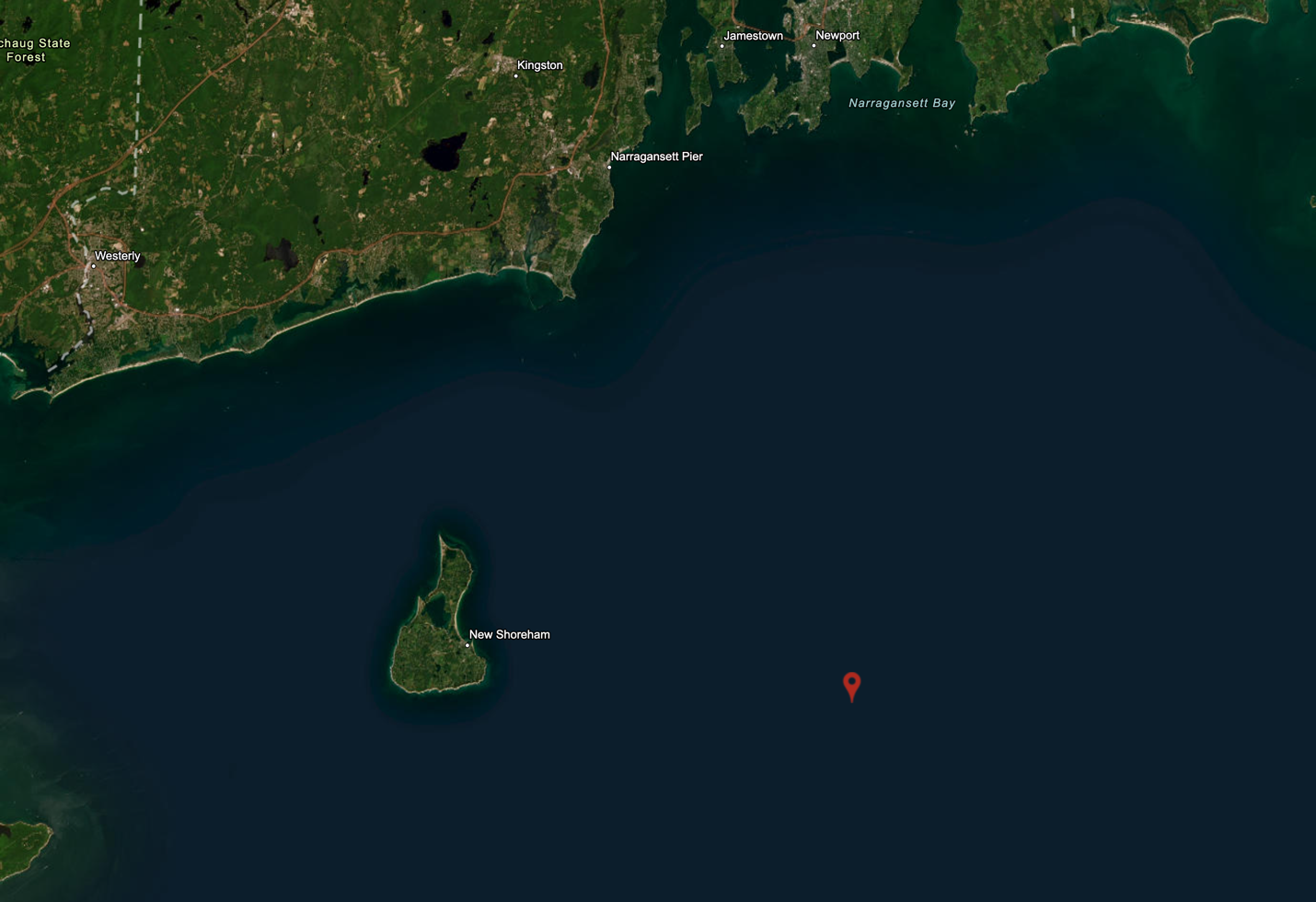


Site 7

Point Judith

Block Island

Rhode Island Sound

**Supplemental Figure 7.** Offshore Site 7, located 25 km southeast of Point Judith in Rhode Island Sound.

A


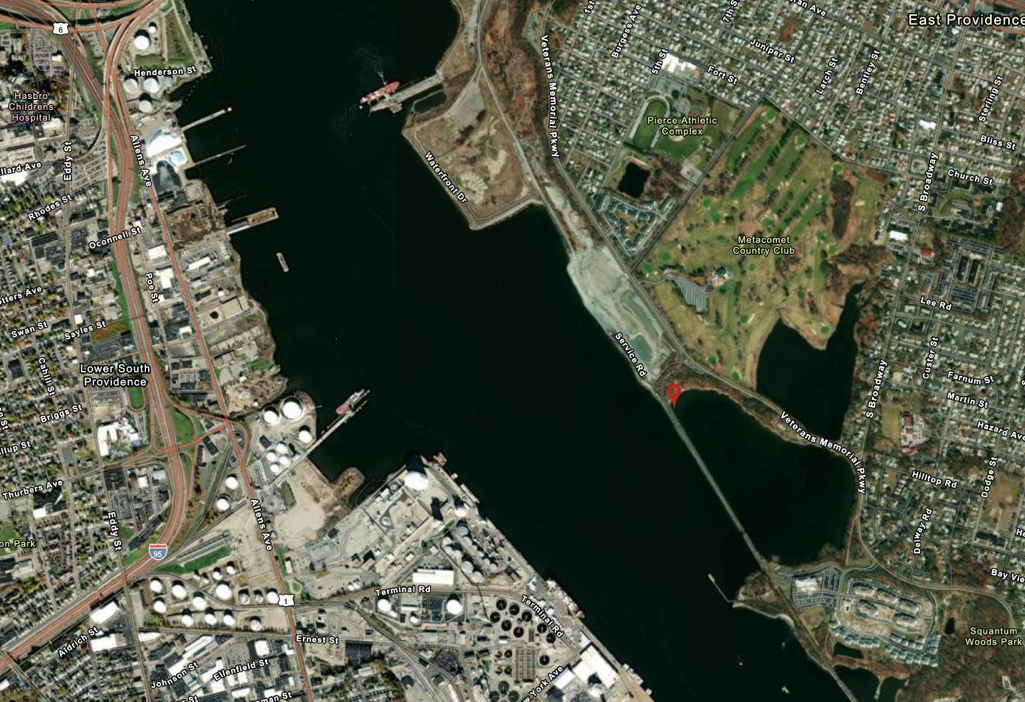


Site M1

B


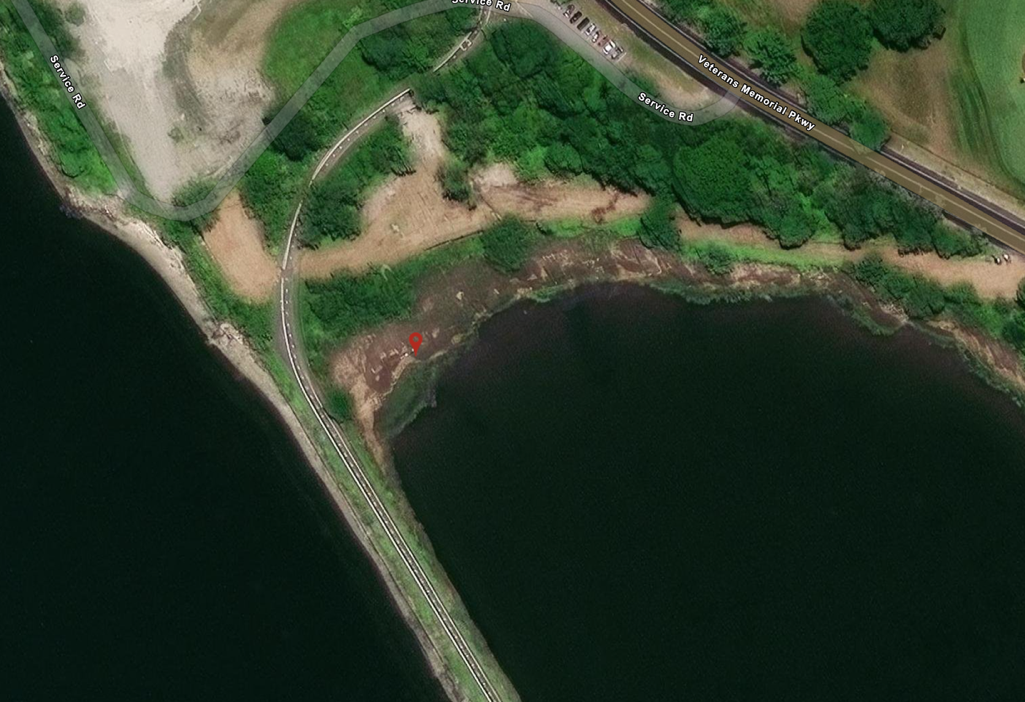


Site M1

**
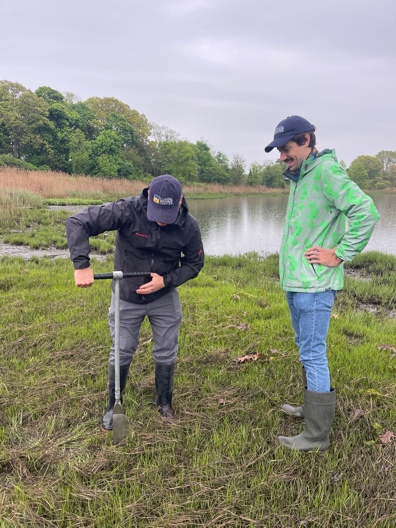
**

C

**Supplemental Figure 8.** Marsh Site M1, located in Watchemocket Cove outside of Providence, RI. Image C shows the marsh coring in action at Site M1.


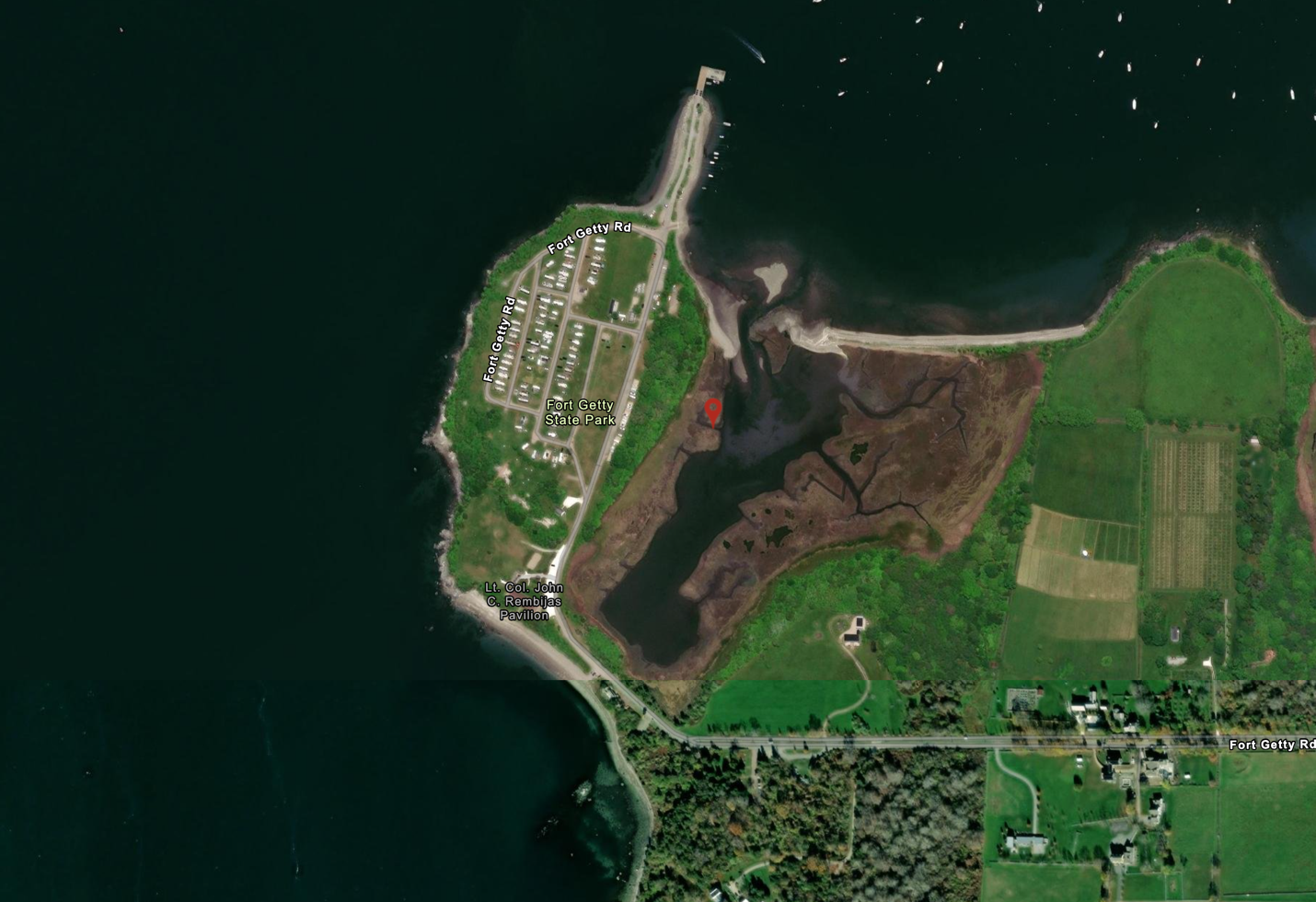


Outer Marsh

Inner Marsh

Site M2

Site M3

A

**
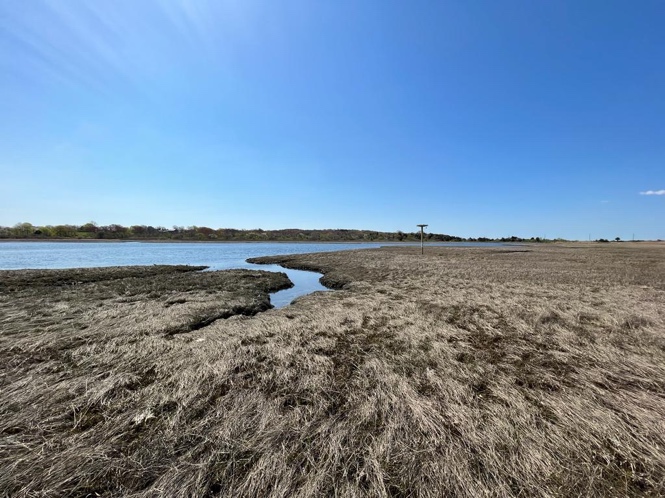

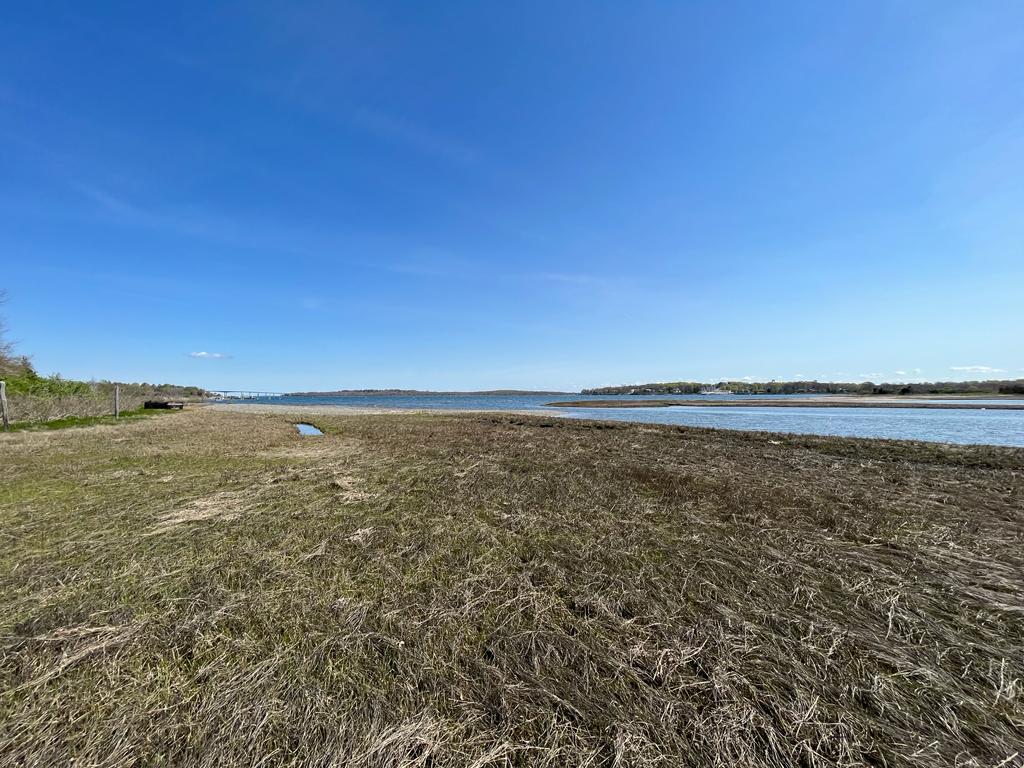
**

B

C

**Supplemental Figure 9.** Marsh Sites M2 and M3, located in Fox Hill Marsh (A). Site M2 was located in the outer marsh (B), while Site M3 was in the inner marsh (C).

**Supplemental Figure 10.** The top 12 plastic polymer spectra (red), compared to their reference library spectra (black), are shown.

**Supplemental Figure 10 (continued).** The top 12 plastic polymer spectra (red), compared to their reference library spectra (black), are shown.

**
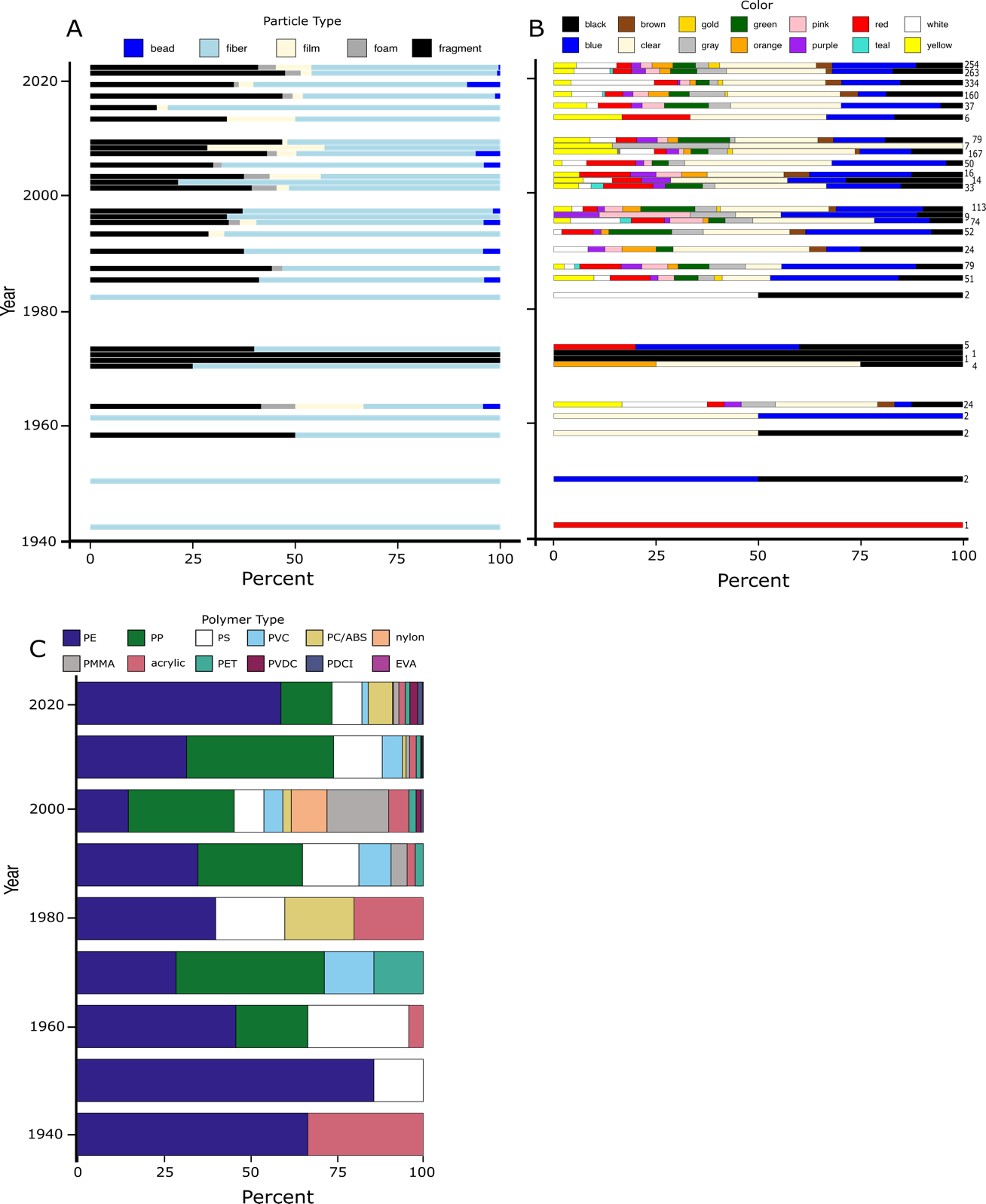
**

**Supplemental Figure 11.** The diversity of microplastic particle morphology, color, and polymer type varies over time in each sediment core. Diversity of particles (A) is highest in the most recent sediment layers. Diversity in color (B) increases dramatically after 1980. The number of particles (n = #) represented by each bar for morphology and color noted on the right of B. Polymer diversity increases over time (C). Polymer type abbreviations are as follows: Polyethylene (PP); polypropylene (PP); polystyrene (PS); polyvinyl chloride (PVC); polycarbonate/acrylonitrile butadiene styrene (PC/ABS); nylon; polymethyl methacrylate (PMMA); acrylic; polyethylene terephthalate (PET); polyvinylidene chloride (PVDC); poly(dicycloundecyl itaconate) (PDCI); ethylene-vinyl acetate (EVA).


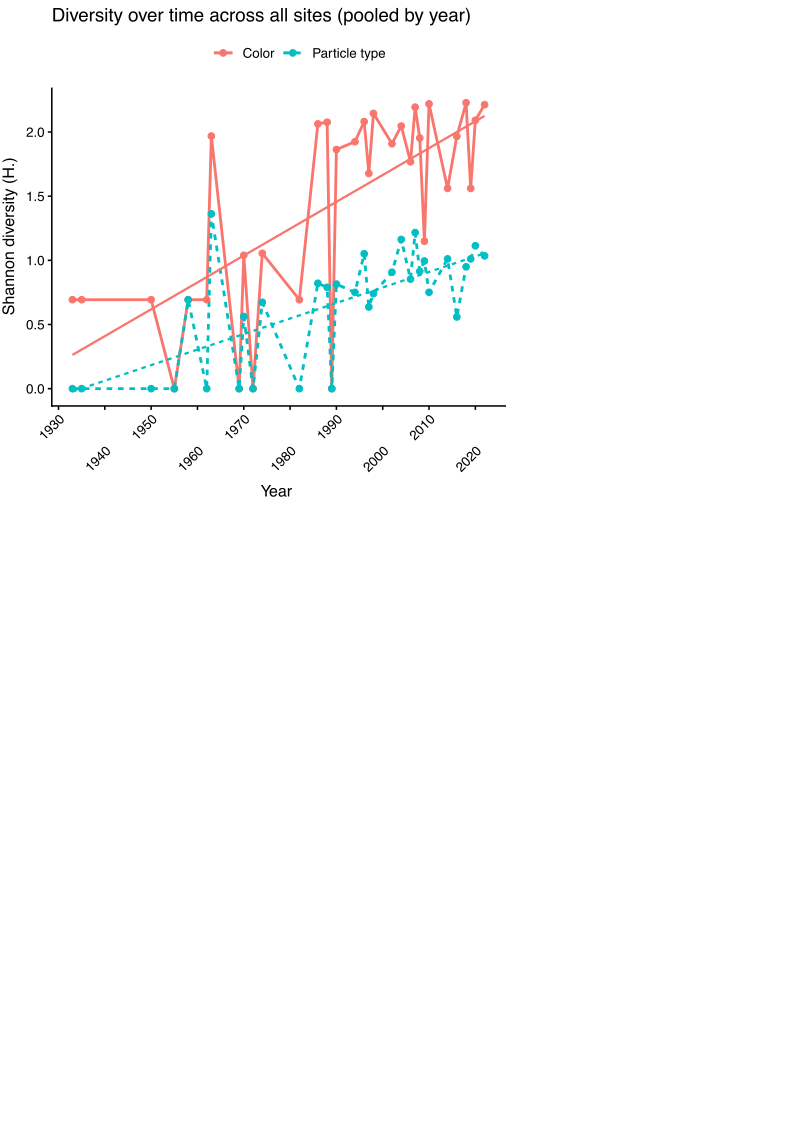


**Supplemental Figure 12.** Increasing Shannon Diversity index for microplastic particle type (morphology) and color from 1930 to the present day.

**
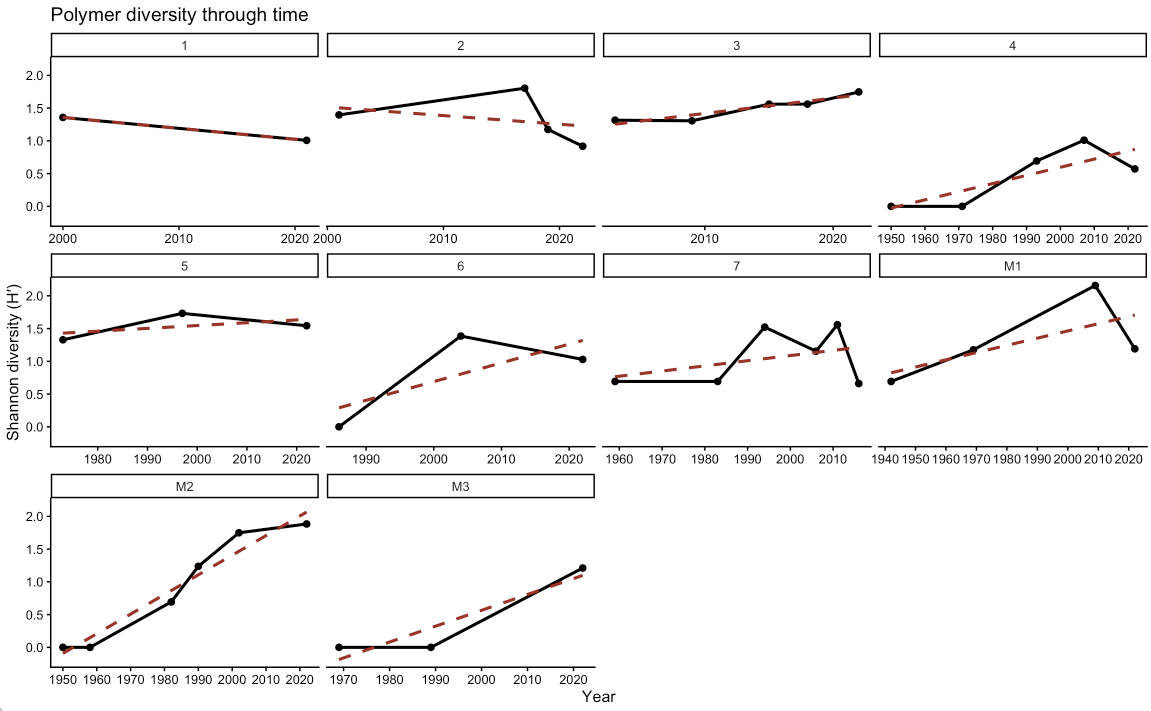
**

**Supplemental Figure 13.** Shannon Diversity (H’) of polymer diversity over time for each site.

**Supplemental Figure 14**. ***(multiple pages)*** Diversity of particle color and particle type, with diversity indices (Shannon Diversity) calculated for each site.

**
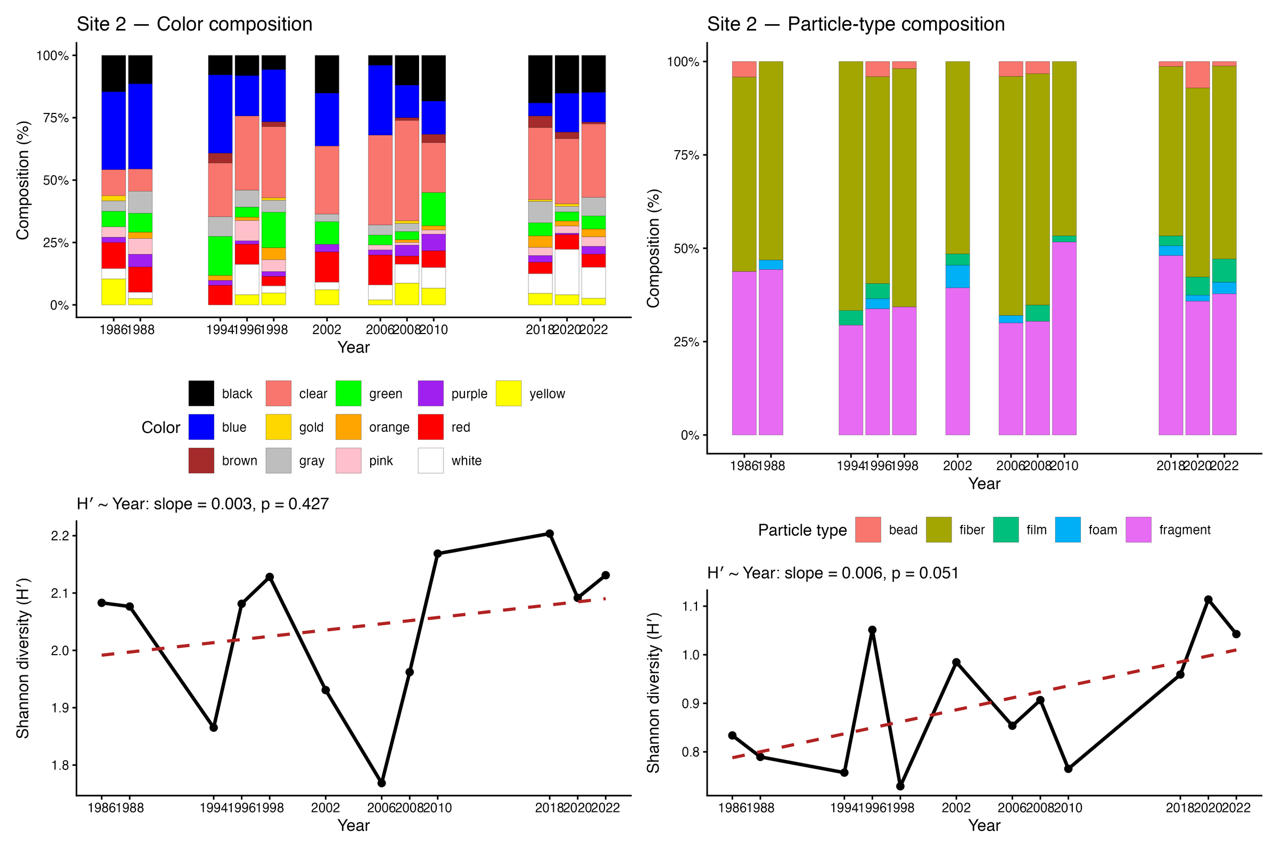
**

**
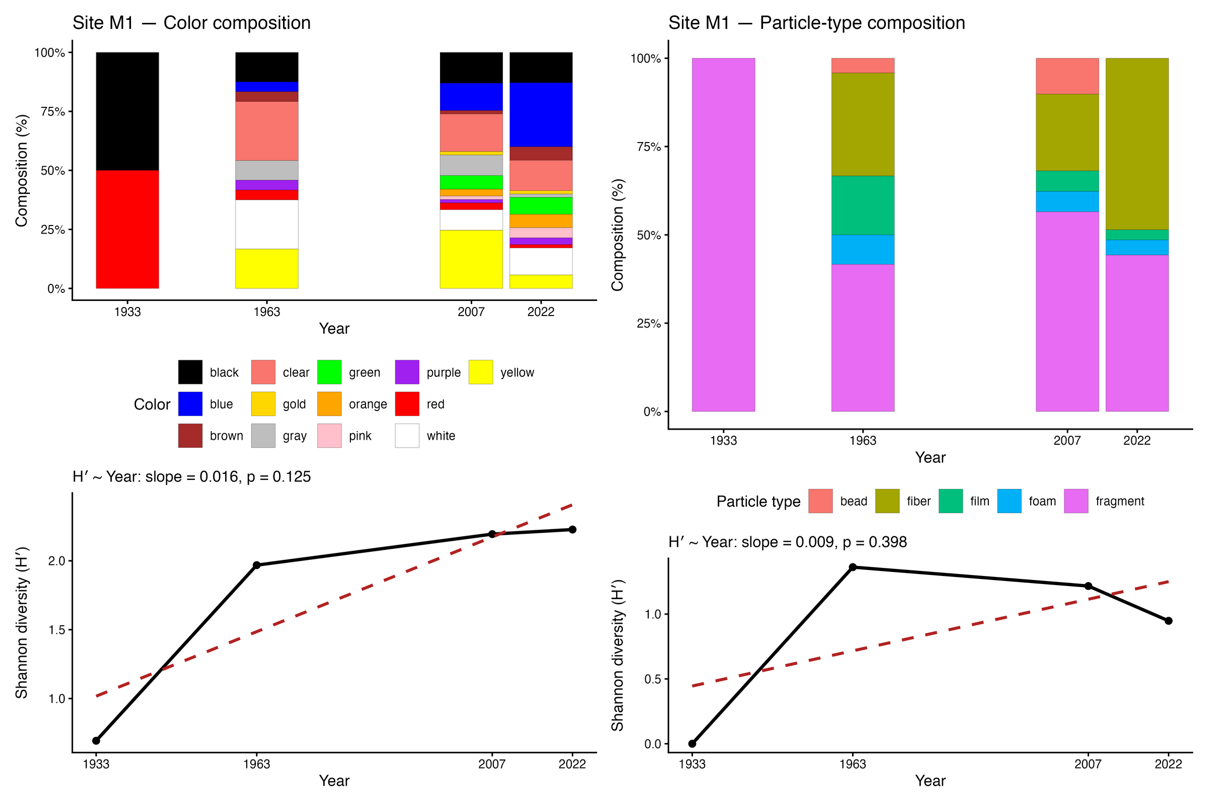
**

**
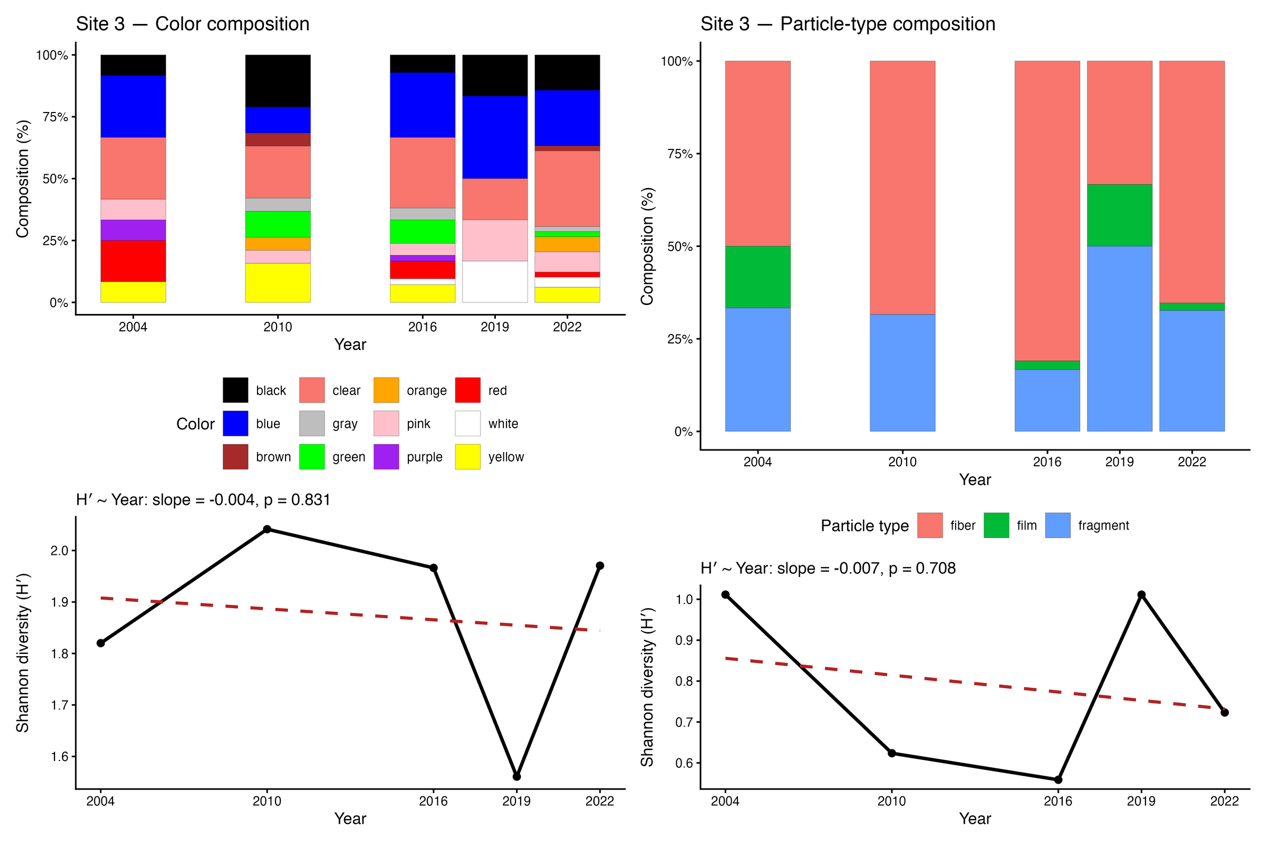
**

**
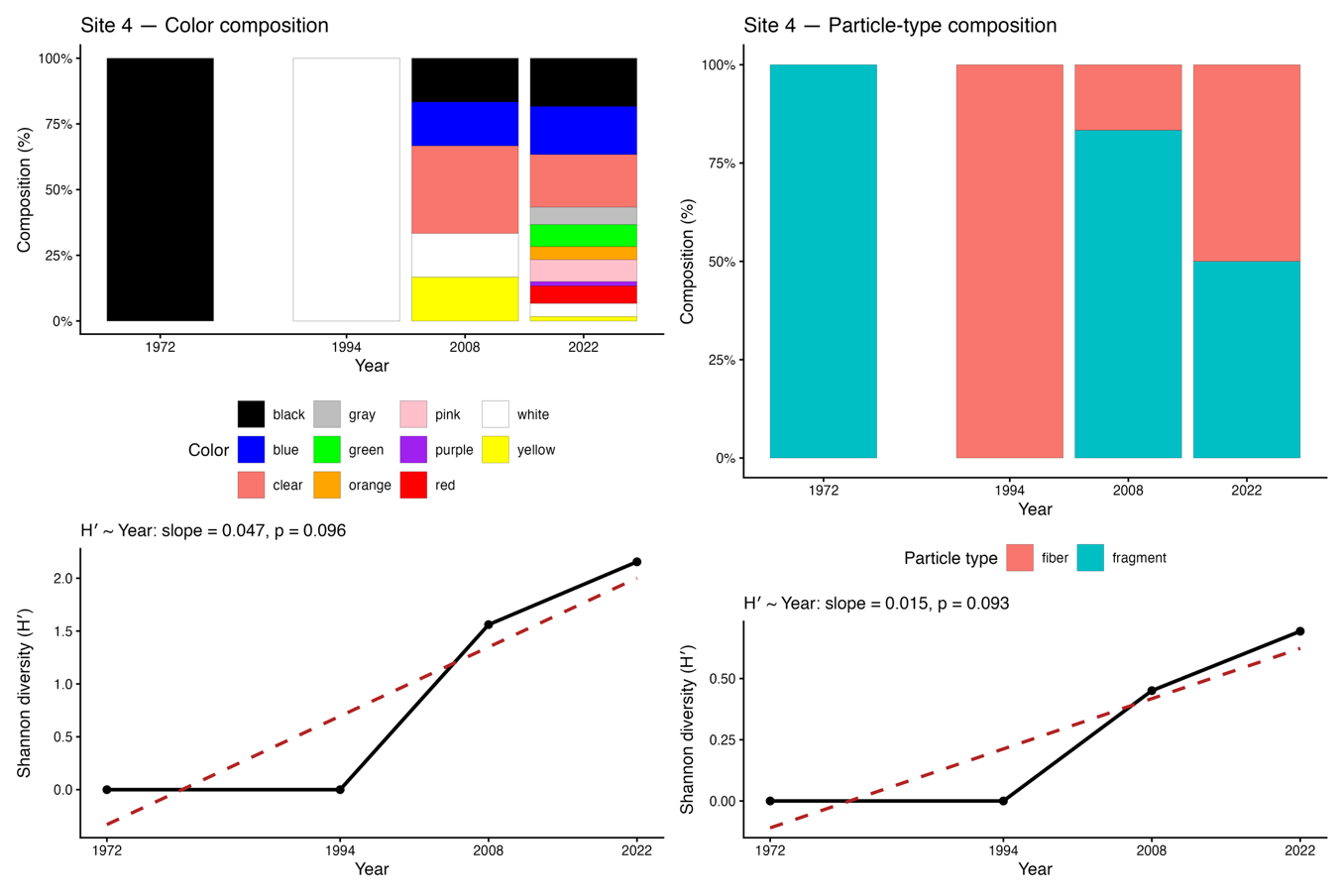
**

**
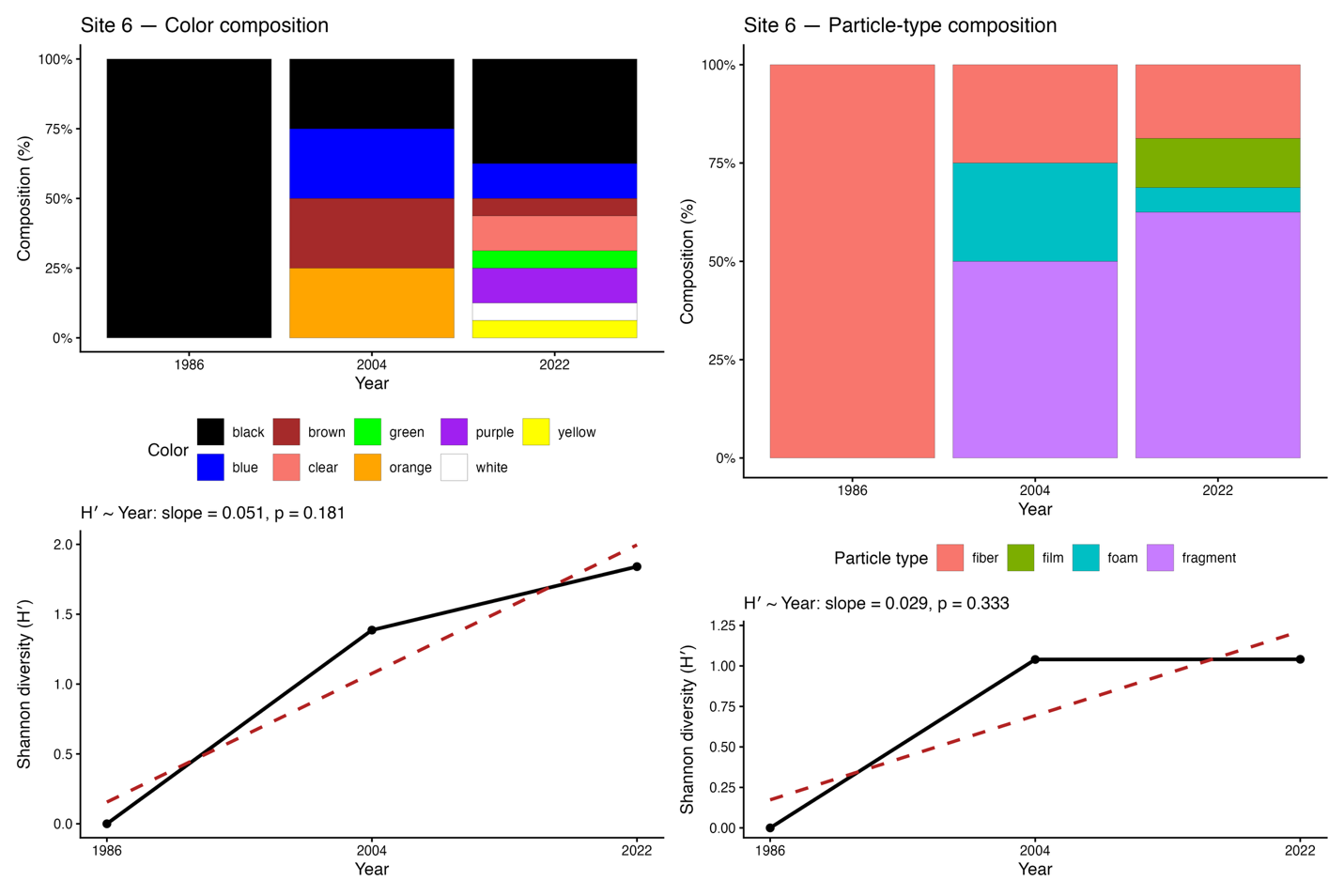

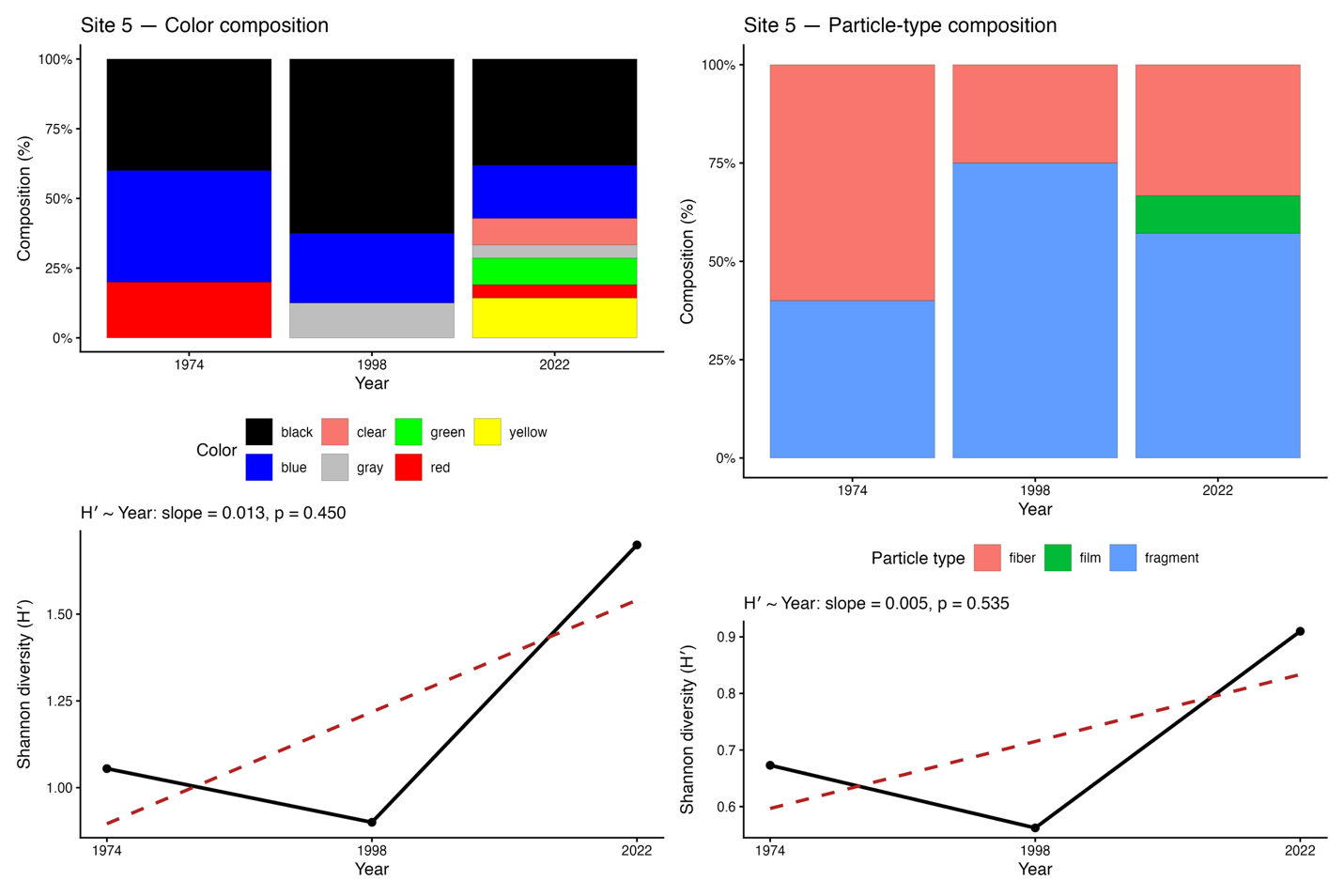
**

**
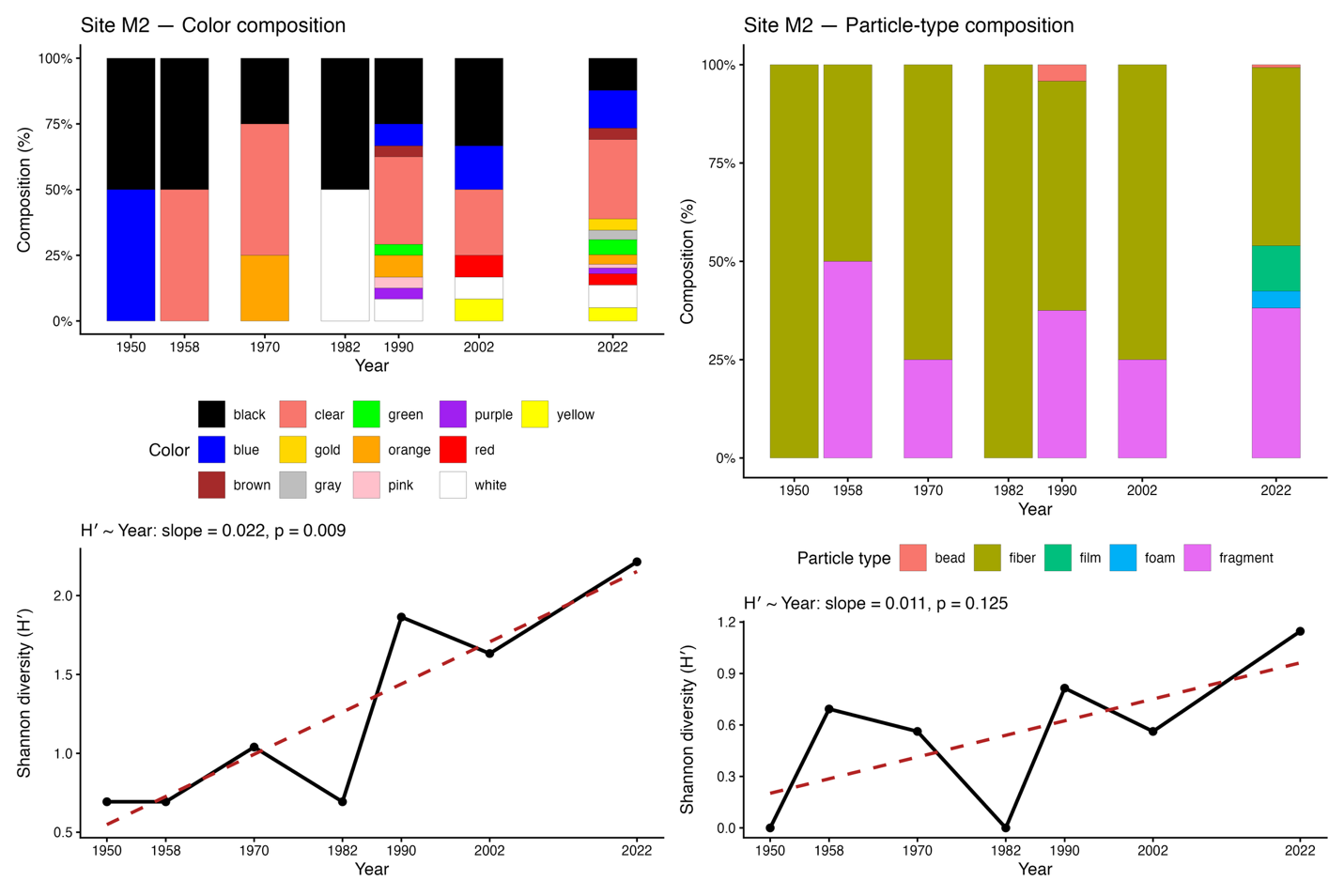

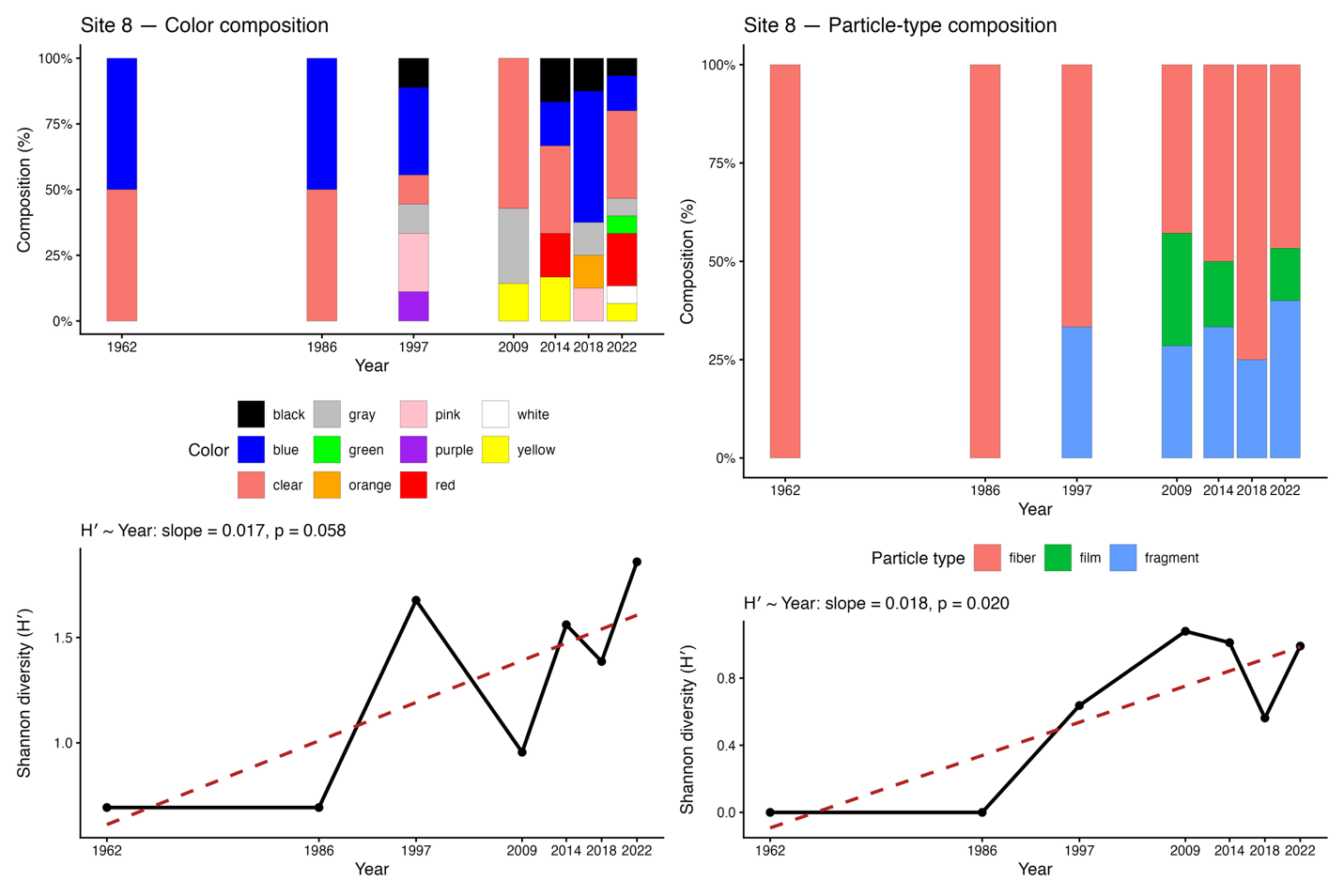
**

Site 7

Site 7

**
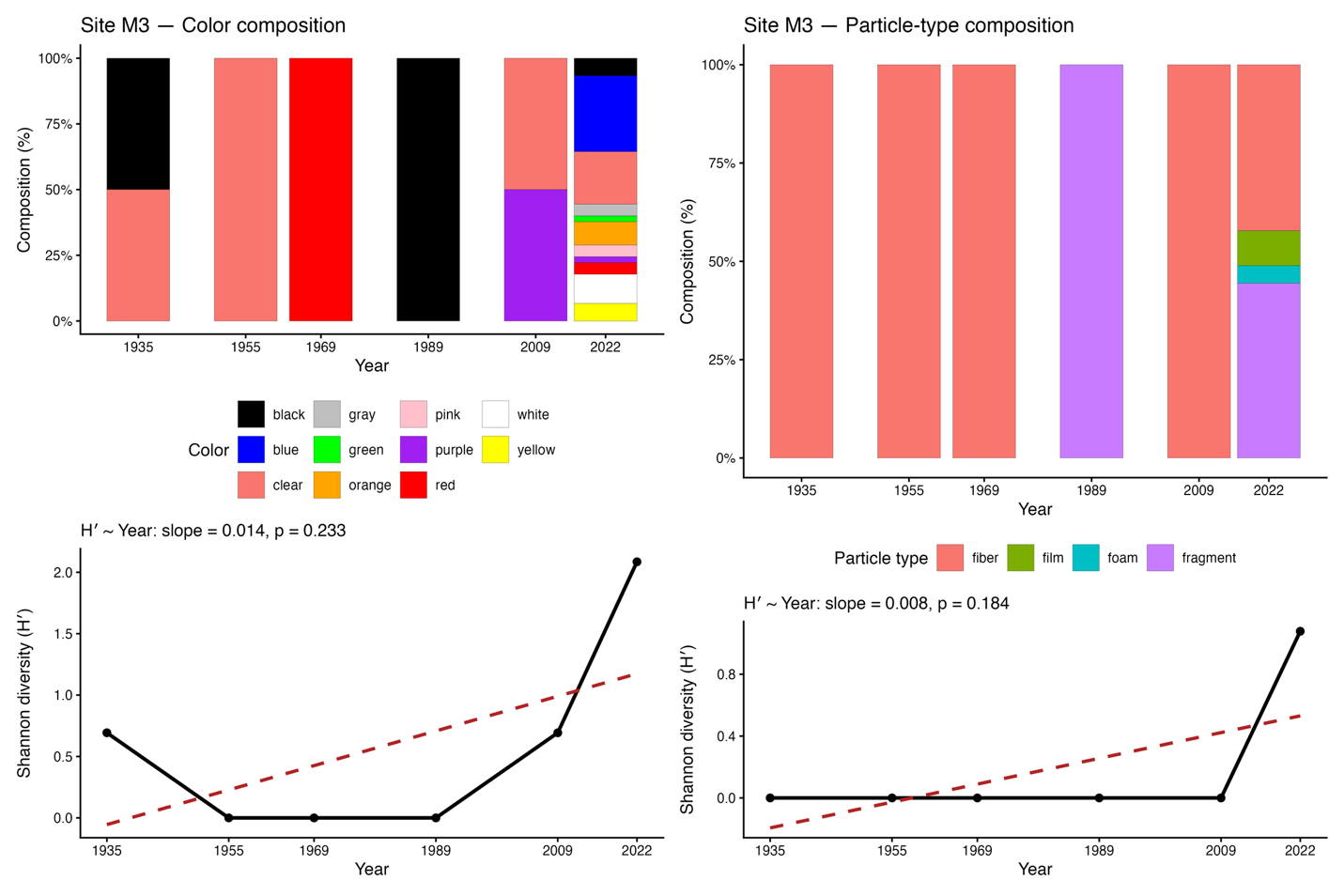
**

**Supplemental Figure 15.** Microplastic concentrations increase over time in Marsh (M1-3) and seafloor (Sites 2-6) cores. The accepted PNEC for exposure of benthic biota to MP pollution is 540 MP particles kg^-1^ (Koelmans et al., 2023; Van Cauwenberghe et al., 2015; Yang et al., 2023). This threshold is denoted by the red dashed line. MP concentrations falling below the threshold are shown in black, and concentrations exceeding the PNEC value are shown in red.

A

**
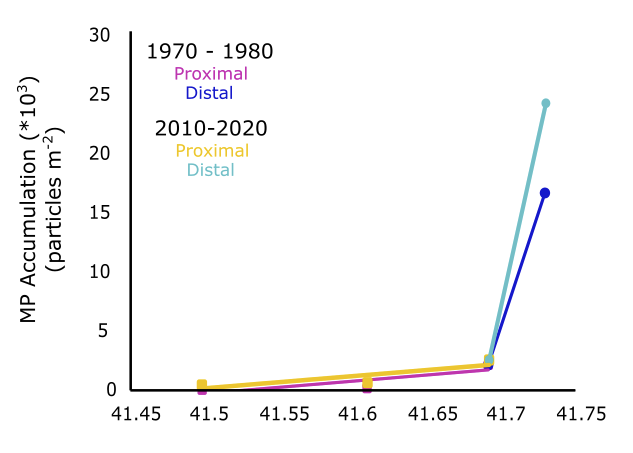
**

B

**Supplemental Figure 16.** Linear fits between latitude and MP accumulation in proximal and distal zones, and for three time periods: 1970-1980 (A), 2010-2020 (A), and 1920-2020 (B).

**Supplemental Figure 17.** Microplastic accumulation rates increased through time, with MP accumulation rates being, on average, 30% lower in the 1970s than in the 2010s. Microplastic samples were taken in deep basins containing finer grain sediments (McMaster, 1960). Microplastic accumulation was highest in the Proximal Zone (warm colors) and lowest in the Distal Zone (cool colors).


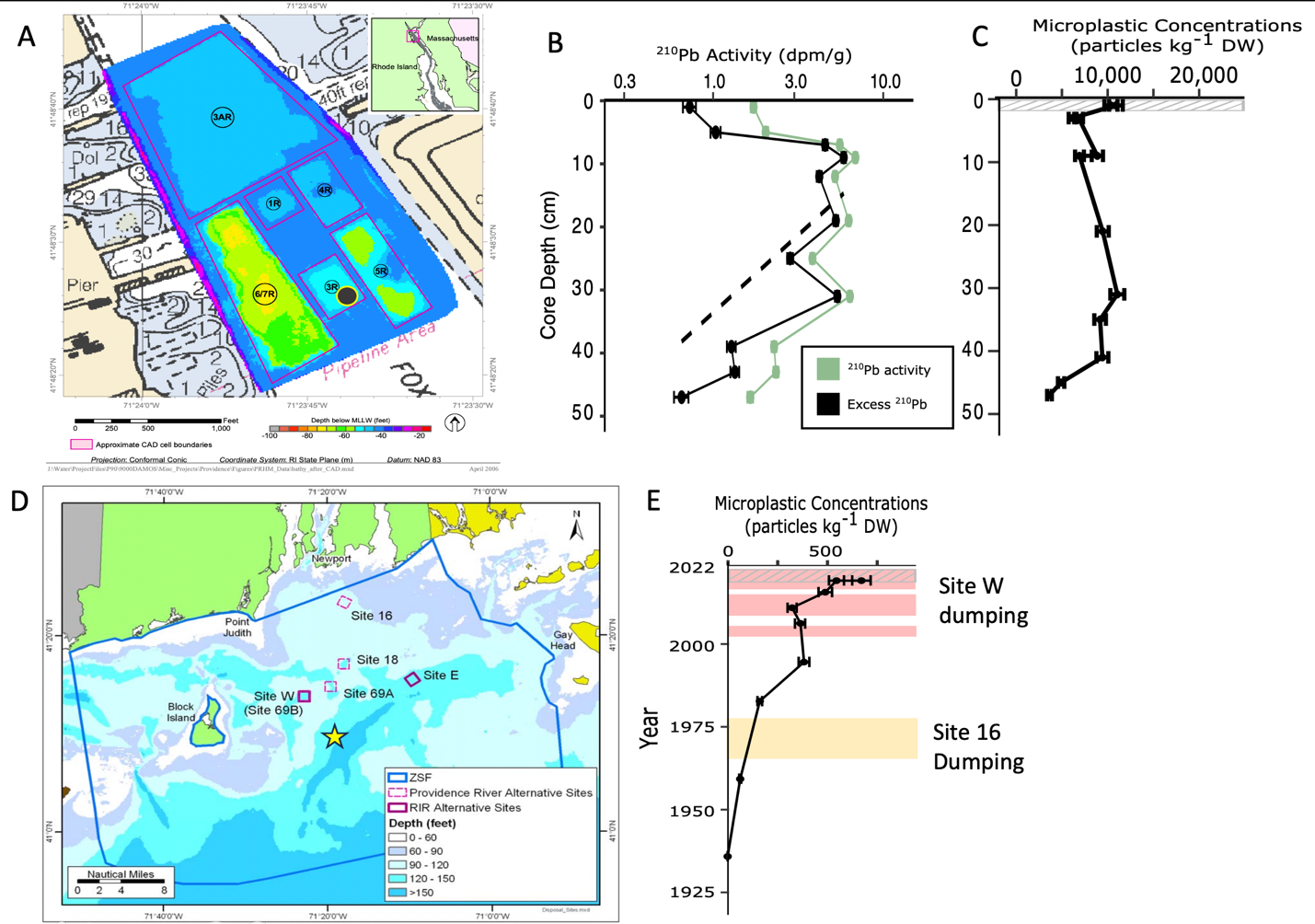


**Supplemental Figure 18.** Sites 1 and 7 are located in anthropogenically-altered/influenced areas. Site 1 (black circle) is located in a confined aquatic disposal (CAD) cell in the Providence River (A; modified from (USACE & INSPIRE Environmental, 2020). The dredging of this area (1971, 1976, 2004) and the creation and subsequent, intermittent filling of the CAD cell (2003 – present) have lead to a highly disturbed site with non-native sediment load pulses, making an age model difficult to construct (B). The high, consistent levels of microplastics in the top 40 cm of the core are consistent with this core representing only recent sediment accumulation (C). Site 7 (yellow star) is located less than 10 km from major offshore dredge sediment disposal sites (D; modified from (USACE, 2021). The timing of these dumpings and the ability for small, low-density particles to travel far distances before being deposited on the seafloor, could explain the high MP concentrations found at Site 7 through time (E).
